# Natural Selection at the Brush-Border: Adaptations to Carbohydrate Diets in Humans and Other Mammals

**DOI:** 10.1093/gbe/evv166

**Published:** 2015-09-11

**Authors:** Chiara Pontremoli, Alessandra Mozzi, Diego Forni, Rachele Cagliani, Uberto Pozzoli, Giorgia Menozzi, Jacopo Vertemara, Nereo Bresolin, Mario Clerici, Manuela Sironi

**Affiliations:** ^1^Bioinformatics, Scientific Institute IRCCS E.MEDEA, Bosisio Parini, Italy; ^2^Dino Ferrari Centre, Department of Physiopathology and Transplantation, University of Milan, Fondazione Ca' Granda IRCCS Ospedale Maggiore Policlinico, Italy; ^3^Department of Physiopathology and Transplantation, University of Milan, Italy; ^4^Don C. Gnocchi Foundation ONLUS, IRCCS, Milan, Italy

**Keywords:** *MGAM*, *SI*, *LCT*, *TREH*, *SLC2A2*, natural selection

## Abstract

Dietary shifts can drive molecular evolution in mammals and a major transition in human history, the agricultural revolution, favored carbohydrate consumption. We investigated the evolutionary history of nine genes encoding brush-border proteins involved in carbohydrate digestion/absorption. Results indicated widespread adaptive evolution in mammals, with several branches experiencing episodic selection, particularly strong in bats. Many positively selected sites map to functional protein regions (e.g., within glucosidase catalytic crevices), with parallel evolution at *SI* (sucrase-isomaltase) and *MGAM* (maltase-glucoamylase). In human populations, five genes were targeted by positive selection acting on noncoding variants within regulatory elements. Analysis of ancient DNA samples indicated that most derived alleles were already present in the Paleolithic. Positively selected variants at *SLC2A5* (fructose transporter) were an exception and possibly spread following the domestication of specific fruit crops. We conclude that agriculture determined no major selective event at carbohydrate metabolism genes in humans, with implications for susceptibility to metabolic disorders.

## Introduction

Diet played an extremely important role in the evolution of mammals and pathways that allow nutrient breakdown and absorption, as well as taste perception, evolved in response to changes in trophic strategies (Karasov et al. 2011). In particular, simple and complex sugars account for a different proportion of energy intake in diverse species and a positive relationship is observed between the dietary intake of carbohydrates and the presence of gut enzymes and transporters necessary for their digestion and absorption (Karasov et al. 2011).

**Fig. 1.—:**
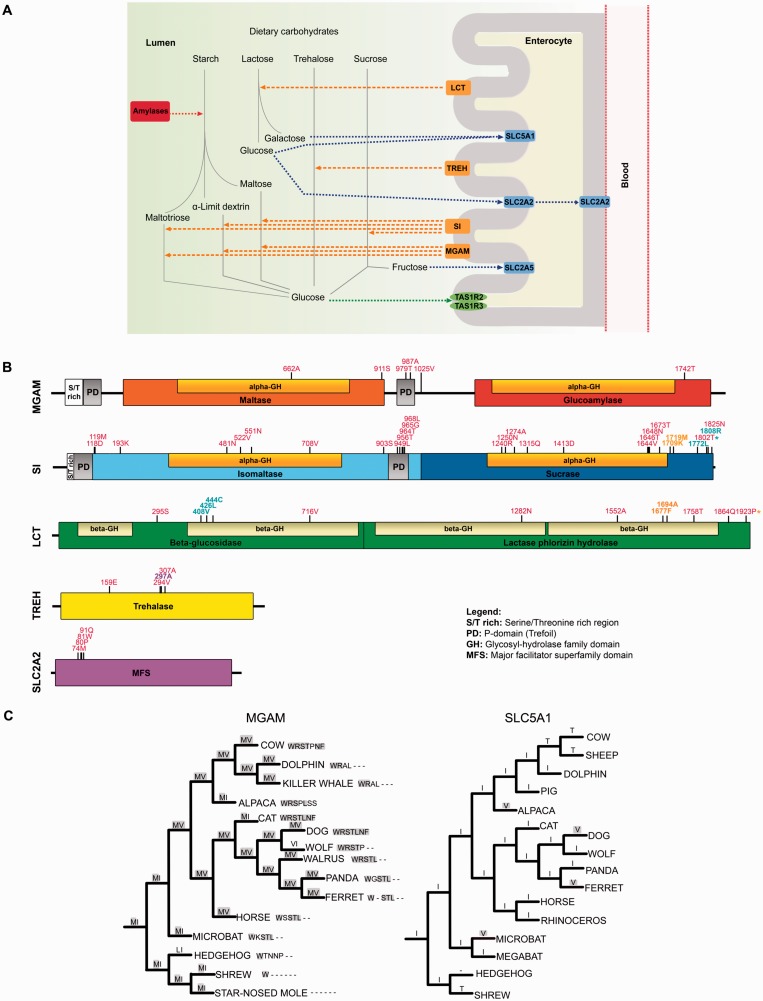
Analyzed genes, protein domain structure, and dog gene analysis. (*A*) The image is modified from KEGG (hsa04967) and gene products are color-coded with enzymes in orange, transporters in blue, and taste receptors in green. Amylases (not included in this study) are shown in red. (*B*) Domain representation of positively selected genes. Sites selected in whole phylogeny are in red; positively selected sites in the human, chimpanzee, and gorilla lineage are in cyan, orange, and violet, respectively. Asterisks denote lineage-specific sites that are also selected in whole phylogeny. Positions refer to the human sequence. (*C*) MGAM and SLC5A1 phylogenetic tree for Laurasiatheria. Amino acid status at positions 797 and 1001, as well as at the seven C-terminal positions is shown for MGAM. Gray shading indicates identity with the dog sequence. Position 244 is reported for SLC5A1.

In humans, culture has paralleled and often affected genetic evolution; in particular, the domestication of plant and animals determined dramatic dietary shifts during the evolution of our species. One of the most prominent signals of positive selection in the genome of European populations is observed at the *LCT* gene, encoding a small intestine brush-border enzyme that catalyzes the hydrolysis of lactose into monosaccharides that can be absorbed (fig. 1*A*) (Tishkoff et al. 2007). Variants that allow *LCT* expression after weaning are strongly selected for in populations that historically relied on animal husbandry (Tishkoff et al. 2007). Likewise, the development of agriculture resulted in starch being an increasingly abundant component in human diets. In our species, duplication of the pancreatic *AMY2* gene originated the salivary amylase gene (*AMY1*), which shows extensive copy number variation (Perry et al. 2007). The number of *AMY1* copies is higher in populations that consume high-starch diets, indicating selection for increasing starch digestion capacity (Perry et al. 2007). Analysis of dog genomes also revealed polymorphic increase in *AMY2B* (pancreatic) copy number during domestication, suggesting that these animals adapted to a diet rich in agricultural refuse (Axelsson et al. 2013; Freedman et al. 2014).

In most mammals amylases catalyze the first step in the digestion of starch; the following reactions occur in the small intestine where, in addition to LCT, three brush-border enzymes, trehalase (TREH), maltase-glucoamylase (MGAM), and sucrase-isomaltase (SI) break down complex sugars into monosaccharides (fig. 1*A* and table 1). These latter are then transported to enterocytes by specialized molecules (SLC5A1, SLC2A2, and SLC2A5), located at the apical brush-border membrane (fig. 1*A* and table 1). In addition to enzymes and transporters, sweet taste receptors (TAS1R2 and TAS1R3) have also been observed at the intestinal brush-border apical membrane in different mammals, where they probably activate gut hormone secretion through glucose sensing (fig. 1*A* and table 1).

**Table 1:** List of the Nine Brush-Border Genes Analyzed and Average Nonsynonymous/Synonymous Substitution Rate Ratio (dN/dS)

Gene Symbol	Aliases	Protein Name	Protein Size (amino acids)	Number of Species	Average dN/dS (95% confidence intervals)
*MGAM*	*MGA, MGAML*	Maltase glucoamylase	1,854	43	0.250 (0.243, 0.257)
*SI*	—	Sucrase isomaltase	1,833	40	0.286 (0.279, 0.293)
*LCT*	*LPH*	Lactase-phlorizin hydrolase	1,934	42	0.263 (0.257, 0.269)
*TREH*	*TREA*	Trehalase	583	43	0.250 (0.240, 0.262)
*SLC2A2*	*GLUT2*	Solute carrier family 2, facilitated glucose transporter member 2	524	46	0.261 (0.249, 0.274)
*SLC5A1*	*NAGT, SGLT1*	Sodium/glucose cotransporter 1	664	46	0.172 (0.165, 0.182)
*SLC2A5*	*GLUT5*	Solute carrier family 2, facilitated glucose transporter member 5	501	42	0.200 (0.191, 0.210)
*TAS1R2*	*GPR71, T1R2, TR2*	Taste receptor type 1 member 2	839	39	0.272 (0.264, 0.281)
*TAS1R3*	*T1R3, TR3*	Taste receptor type 1 member 3	852	29	0.238 (0.230, 0.247)

In line with the central role of starch metabolism in humans and other mammals, the *MGAM* and *SLC5A1* loci were targeted by natural selection in dogs (Axelsson et al. 2013). In humans, signals of selection at genes involved in starch and sucrose metabolism have been detected for populations that rely on roots and tubers as staple foods (Hancock et al. 2010). Nonetheless, the evolution of brush-border carbohydrate metabolic genes has never been analyzed in detail. Herein, we use both inter- and intraspecies comparisons to analyze the evolution of these nine genes in mammals and human populations. For the interspecies analyses, we focused on coding regions by applying different methods to assess whether brush-border carbohydrate metabolic genes were targets of either pervasive or episodic positive selection. In this context, positive selection is defined by a faster rate of accumulation of nonsynonymous (amino acid-replacing) compared with synonymous (nonamino acid-replacing) substitutions, a pattern that may involve only a limited number of sites in a protein. If the selective pressure acted on a limited number of lineages in a phylogeny, it is said to be “episodic.” As for intraspecies analyses, we focused on human populations and integrated information concerning archaic hominins: this allowed testing of specific hypotheses as to when adaptive alleles at genes involved in sugar metabolism arose or spread. In this case, we analyzed both coding and noncoding regions and we define positive selection as the frequency increase in a population of a beneficial variant/haplotype (also referred to as selective sweep). The general underlying premise for this study is that natural selection acts on functional genetic variants with a phenotypic effect. Therefore, evolutionary analysis can provide information on the location and nature of adaptive changes that modulate phenotypic diversity in humans and other mammals.

## Materials and Methods

Algorithms, programs, and tests applied for all analyses are summarized in supplementary table S1, Supplementary Material online.

### Evolutionary Analysis in Mammals

Mammalian sequences genes were retrieved from the NCBI database (as of January 7, 2015) (supplementary table S2, Supplementary Material online). Mammalian orthologs of human brush-border genes were included only if they represented one-to-one orthologs as reported in the EnsemblCompara GeneTrees (Vilella et al. 2009). The *MGAM* gene may have undergone domain duplications in some mammals (Naumov 2007). Although all the sequences we obtained from NCBI were comparable in size to the human sequence, we cannot exclude annotation errors and, therefore, aligning of paralogous domains. However, we note that, even in this case, our results would not be significantly affected because the methods we used to detect positive selection are equally applicable to paralogous and orthologous regions (Bielawski and Yang 2003).

DNA alignments were performed using the RevTrans 2.0 utility (Wernersson and Pedersen 2003), which uses the protein sequence alignment as a scaffold for constructing the corresponding DNA multiple alignment. Alignment uncertainties were removed using trimAl (automated1 mode) (Capella-Gutierrez et al. 2009). Alignments were checked by hand before running selection tests.

Recombination may yield false positive results when tests of positive selection are applied (Anisimova et al. 2003). This is because most methods used to infer positive selection assume that the phylogenetic tree and branch lengths are constant across all sites in the alignment, a tenet that is invalid in the presence of recombination. We thus screened all alignments for the presence of recombination breakpoints (the locations where recombination events occur in the alignments) using GARD (genetic algorithm recombination detection) (Kosakovsky Pond et al. 2006). No evidence of recombination was detected for *LCT, SLC2A2*, and *TAS1R2*, whereas breakpoints were detected for the remaining genes.

SLAC (single likelihood ancestor counting) was applied to calculate the average nonsynonymous substitution/synonymous substitution rate (dN/dS) for the nine genes (Kosakovsky Pond and Frost 2005). To detect positive selection, we used the site models implemented in PAML (Yang 1997, 2007); NSsite models that allow (M2a, M8) or disallow (M1a, M7) sites to evolve with dN/dS >1 were fitted to the data with two models of equilibrium codon frequencies: the F3x4 model (codon frequencies estimated from the nucleotide frequencies in the data at each codon site) and the F61 model (frequencies of each of the 61 non-STOP codons estimated from the data) (supplementary table S3, Supplementary Material online). These analyses were performed either for whole gene alignments or independently for subregions defined in accordance with the recombination breakpoints. In these latter cases, Bonferroni correction for multiple tests was applied to the maximum-likelihood ratio tests (LRTs) *P* values (supplementary table S3, Supplementary Material online). Trees were generated by maximum-likelihood using the program PhyML (Guindon et al. 2009). Whenever maximum-likelihood trees showed differences (always minor) from the accepted mammalian phylogeny, analyses were repeated using the accepted tree, and the same results were obtained in all cases (not shown). Sites under selection with the M8 model were identified using Bayes Empirical Bayes (BEB) analysis with a significance cutoff of 0.90 (Anisimova et al. 2002; Yang et al. 2005). For MEME (mixed effects model of evolution) (Murrell et al. 2012) the default cutoff of 0.10 was used.

To explore possible variations in selective pressure among different mammals for the five positively selected genes, we tested whether models that allow dN/dS to vary along branches had significant better fit to the data than models that assume one same dN/dS across the entire phylogeny (Yang and Nielsen 1998). This condition was verified for all genes (supplementary table S4, Supplementary Material online).

To identify specific branches with a proportion of sites evolving with dN/dS > 1, we used BS-REL (Branch Site-Random Effects Likelihood) (Kosakovsky Pond et al. 2011). This method implements branch-site models that simultaneously allow dN/dS variation across branches and sites. One advantage of BS-REL is that it requires no prior knowledge about which lineages are of interest (i.e., are more likely have experienced episodic diversifying selection). Branches identified using this approach were cross-validated using the branch-site LRTs from codeml (the so-called modified model A and model MA1, “test 2”) (Zhang et al. 2005). In this test, branches are divided a priori into foreground (those to be analyzed for positive selection) and background lineages, and a LRT is applied to compare a model that allows positive selection on the foreground lineages with a model that does not allow such positive selection. A false discovery rate correction was applied to account for multiple hypothesis testing (i.e., we corrected for the number of tested lineages), as suggested (Anisimova and Yang 2007). MEME and BEB analysis from MA (with a cutoff of 0.90) were used to identify sites that evolve under positive selection on specific lineages (supplementary table S5 and figs. S1 and S2, Supplementary Material online).

Ancestral site reconstruction was obtained through the DataMonkey sever by ASR (Ancestral Sequence Reconstruction) utility, which implements three different methods (Delport et al. 2010).

GARD, MEME, SLAC, and BS-REL analyses were performed either through the DataMonkey server (Delport et al. 2010) or run locally (through HyPhy) (supplementary table S1, Supplementary Material online).

### Population Genetics-Phylogenetics Analysis

Data from the Pilot 1 phase of the 1000 Genomes Project were retrieved from the dedicated website (1000 Genomes Project Consortium et al. 2010). Single nucleotide polymorphism (SNP) genotype information for 25 unrelated chimpanzees and 27 unrelated gorillas were retrieved from (Prado-Martinez et al. 2013). Coding sequence information was obtained for the nine genes and the ancestral sequence was reconstructed by parsimony from the human, chimpanzee, orangutan, and macaque sequences. Analyses were performed with gammaMap (Wilson et al. 2011).

For gammaMap analysis, we assumed θ (neutral mutation rate per site), k (transitions/transversions ratio), and T (branch length) to vary among genes following log-normal distributions. For each gene we set the neutral frequencies of non-STOP codons (1/61) and the probability that adjacent codons share the same selection coefficient (*P* = 0.02). For selection coefficients, we considered a uniform Dirichlet distribution with the same prior weight for each selection class. For each gene, we run 10,000 iterations with thinning interval of ten iterations.

### Population Genetics Analyses

A set of programs was developed to retrieve genotypes from the 1000 Genomes Pilot Project MySQL database (1000 Genomes Project Consortium et al. 2010) and to analyze them according to selected regions/populations. These programs were developed in C++ using the GeCo++ (Cereda et al. 2011) and the libsequence (Thornton 2003) libraries. Genotype information was obtained for the nine brush-border genes. In order to obtain a control set of approximately 1,000 genes to use as a reference set, we initially selected 1,200 genes by random sampling of those included in the RefSeq list. For these genes we retrieved orthologous regions in the chimpanzee, orangutan, or macaque genomes (outgroups) using the LiftOver tool; genes showing less than 80% human-outgroup aligning bases were discarded. This originated a final set of 987 genes, hereafter referred to as control set. Compared with the control set, no brush-border gene was exceptional in terms of recombination rate and none (with the exclusion of *TAS1R3*, which displayed no selection signature) had unusually high GC content, which may bias selection inference (Pollard et al. 2006) (supplementary fig. S3, Supplementary Material online).

Nucleotide diversity over whole gene regions was measured as π (Nei and Li 1979) and θ_W_ (Watterson 1975). DH (Fay and Wu 2000; Zeng et al. 2006) was also calculated in 5 kb sliding windows moving with a step of 500 bp. Sliding window analyses have an inherent multiple testing problem that is difficult to correct because of the nonindependence of windows. In order to partially account for this limitation, we applied the same procedure to the control gene set, and the distribution of DH was obtained for the corresponding windows. This allowed calculation of the fifth percentile and visualization of regions below this threshold.

*F*_ST_ (Wright 1950) and the DIND (derived intra-allelic nucleotide diversity) test (Barreiro et al. 2009) were calculated for all SNPs mapping to the control and brush-border gene sets. Because *F*_ST_ values are not independent from allele frequencies, we binned variants based on their MAF (minor allele frequency, 50 classes) and calculated the percentiles distributions for each MAF class. As for the DIND test, we calculated statistical significance by obtaining an empirical distribution of DIND-DAF (derived allele frequency) value pairs for variants located within control genes. Specifically, DIND values were calculated for all SNPs using a constant number of 40 flanking variants (20 up- and down-stream). The distributions of DIND-DAF pairs for Yoruba (YRI), Europeans (CEU), and Chinese plus Japanese (CHBJPT) was binned in DAF intervals (100 classes) and for each class the percentiles distributions were calculated. As suggested previously (Barreiro et al. 2009), for values of iπ_D_= 0 we set the DIND value to the maximum obtained over the whole data set plus 20. Due to the nature of low-coverage data, for low DAF values most iπ_D_ resulted equal to 0 (i.e., the 95th percentile could not be calculated); thus, we did not calculated DIND in these ranges and we consequently cannot detect selection acting on low frequency derived alleles.

For the DIND test, an approach based on coalescent simulations was also applied to assess statistical significance. In particular, coalescent simulations were performed using the cosi package (Schaffner et al. 2005) with 2,000 iterations. Simulations were conditioned on mutation and recombination rates, and on a region length of 20,000 bp. We simulated demographic patterns using parameters for YRI, CEU, and CHBJPT as described in Grossman et al. (2010) with a data thinning procedure that improves fitting to the 1000 Genomes empirical data (Engelken et al. 2014). Estimates of the population recombination rate parameter ρ were obtained from UCSC table browser.

## Results

### Most Brush-Border Carbohydrate Digestion/Absorption Genes Evolve Adaptively in Mammals

We analyzed the evolutionary history of genes involved in carbohydrate metabolism. These were selected on the basis of KEGG (Kyoto Encyclopedia of Genes and Genomes) pathway “carbohydrate digestion and absorption” (hsa04973) with the inclusion of brush-border proteins only and the addition of *TREH* (GO:0044245, polysaccharide digestion) (fig. 1*A* and table 1). We obtained coding sequence information from public databases. Except for *TAS1R3*, at least 39 species were available for each gene (table 1 and supplementary table S2, Supplementary Material online). We first calculated the average nonsynonymous substitution/synonymous substitution rate (dN/dS) for the nine genes: in all cases dN/dS was much lower than 1 (table 1), indicating a major role for purifying selection in shaping genetic diversity. Although constraints on protein function and structure often result in purifying selection being the primary force that shapes diversity at coding sequences, diversifying selection might involve specific sites or domains. To test this possibility, we applied maximum-LRTs implemented in the codeml program (Yang 2007) after accounting for the presence of recombination. Specifically, we compared models of gene evolution that allow (NSsite models M2a and M8, positive selection models) or disallow (NSsite models M1a and M7, null models) a class of codons to evolve with dN/dS >1. To assure reliability, different codon substitution models were used (supplementary table S3, Supplementary Material online). Results indicated that five brush-border genes were targeted by positive selection in mammals (fig. 1*B* and supplementary table S3, Supplementary Material online). In order to identify specific sites subject to positive selection, we applied the BEB analysis (Yang et al. 2005), which calculates the posterior probability that each codon is from the site class of positive selection (under model M8). An additional method, the MEME (Murrell et al. 2012) was also applied. MEME allows the distribution of dN/dS to vary from site to site and from branch to branch at a site, therefore allowing the detection of both pervasive and episodic positive selection; the method has been shown to have more power than methods that assume constant dN/dS across lineages (Murrell et al. 2012). To be conservative, only sites detected using both BEB and MEME were considered targets of positive selection (fig. 1*B*); their functional implications are analyzed below.

### Different Selective Pressure among Lineages

We next explored possible variations in selective pressure among different mammals for the five positively selected genes (fig. 1*B*, supplementary tables S4 and S5 and figs. S1 and S2, Supplementary Material online).

*SI* showed the strongest evidence of episodic selection: several positively selected residues were identified for rodents and bats, with microbat also showing positive selection at *MGAM* (supplementary table S5 and fig. S2, Supplementary Material online). Interestingly, microbat and platypus, the only two lineages that experienced episodic selection at *TREH* (supplementary table S5 and fig. S1, Supplementary Material online) have a diet that includes trehalose, as these animals feed on insects and crustaceans, respectively.

It was recently suggested that *MGAM* and *SLC5A1* were positively selected in dog. The putative adaptive coding changes are present in the dog reference genome (a boxer) and are accounted for by position M797 and V1001 (dog residues) in MGAM, where a two amino acid C-terminal extension was also noted (Axelsson et al. 2013). Although we did not find evidence of positive selection for any of the analyzed genes in dog (supplementary table S5 and figs. S1 and S2, Supplementary Material online), we analyzed these residues by taking into account the known phylogeny of mammals and by ancestral state reconstruction at internal nodes (this was not feasible for the C-terminal extension). As shown in figure 1*C*, dogs share the M797 and V1001 residues with several related species and these amino acids represent the ancestral state at most nodes. Inference on the C-terminal extension was more difficult, due to extensive variability in this region; dog shares the two amino acids extension with cat, cow, and alpaca, although with minor differences in these two latter species (fig. 1*C*). A similar analysis for the *SLC5A1* putatively selected site (V244) (Axelsson et al. 2013) indicated frequent substitutions at this position, with valine being shared by dog, ferret, and other species (fig. 1*C*). Calculation of dN/dS for this position in the whole phylogeny indicated a value of 1.19, close to selective neutrality.

### Several Positively Selected Sites Impinge on Functional Protein Regions

**Fig. 2.—:**
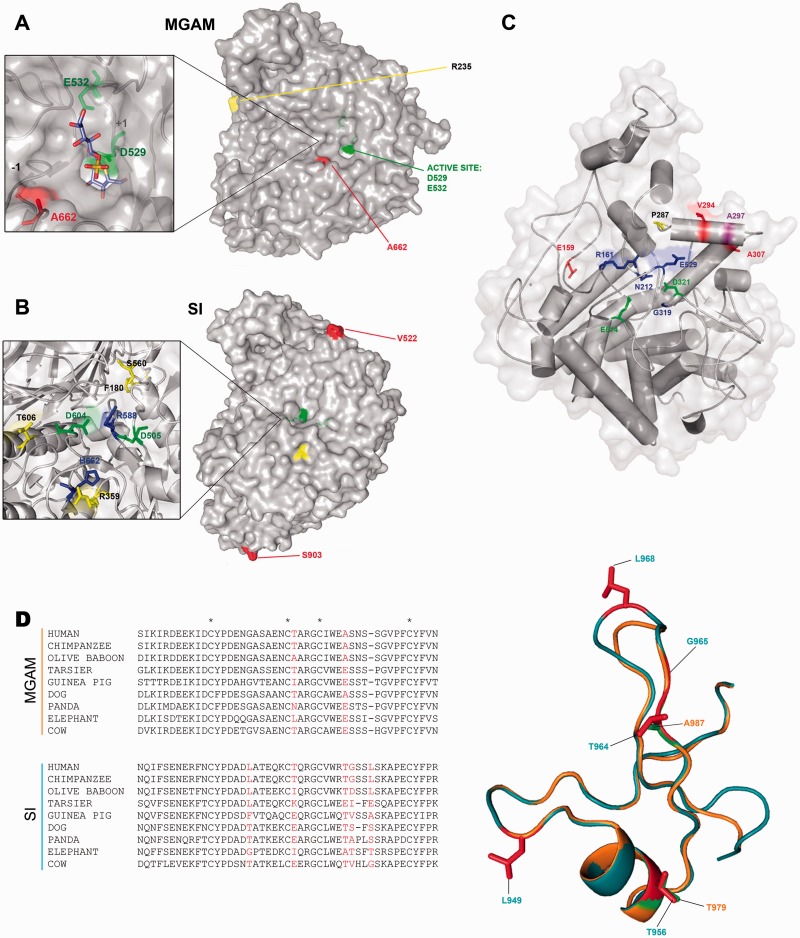
Three dimensional mapping of selected sites. Surface representation of MGAM maltase domain (PDB: 3L4V) in complex with kotalanol (blue stick) (*A*) and SI isomaltase domain (PDB: 3LPP) (*B*). Catalytic crevices are shown in the enlargements; color codes as follows: red, positively selected sites in the whole phylogeny; yellow, lineage-specific sites; orange, and cyan, positively selected sites in the chimpanzee and human lineages, respectively; green, catalytic residues; blue, amino acids involved in ligand binding (Sim et al. 2010). (*C*) Mapping of positively selected sites onto the TREH structure; color codes are as above; violet: positively selected residues in gorilla. (*D*) Multiple alignment of MGAM and SI trefoil domains for a few of representative mammalian species; positively selected sites (whole phylogeny) are in red. Asterisks indicate conserved cysteine residues. The structural superimposition of trefoil domains of MGAM (orange) (PDB code: 3TON) and SI (light blue) (Protein Model Portal code: P14410, Model 2) is also shown. Positively selected sites on whole phylogeny are represented as sticks, green for SI and red for MGAM.

We detected one positively selected site in the maltase domain of MGAM (A662, fig. 1*B*), which is in close spatial proximity to the active site (Sim et al. 2010) (fig. 2*A*). Similarly, in the SI isomaltase subunit some lineage-specific positively selected sites were found to be located in nearby the substrate-binding and active sites (fig. 2*B*, supplementary table S5, Supplementary Material online) (Sim et al. 2010). As for TREH, two of the selected sites we identified, E159 (whole phylogeny) and P287 (microbat) are also in proximity to residues involved in substrate binding (fig. 2*C* and supplementary table S5, Supplementary Material online).

A part from these sites, most selected residues in MGAM and SI are surface-exposed, with some of them defining continuous surface patches (supplementary fig. S4, Supplementary Material online). Moreover, a considerable proportion of positively selected sites maps to the trefoil or P domains (PD, fig. 1*B*). The superimposition of the two PDs revealed that the two positively selected sites of MGAM (T979, A987) correspond to T956 and T964, which are positively selected in SI (fig. 2*D*).

**Fig. 3.—:**
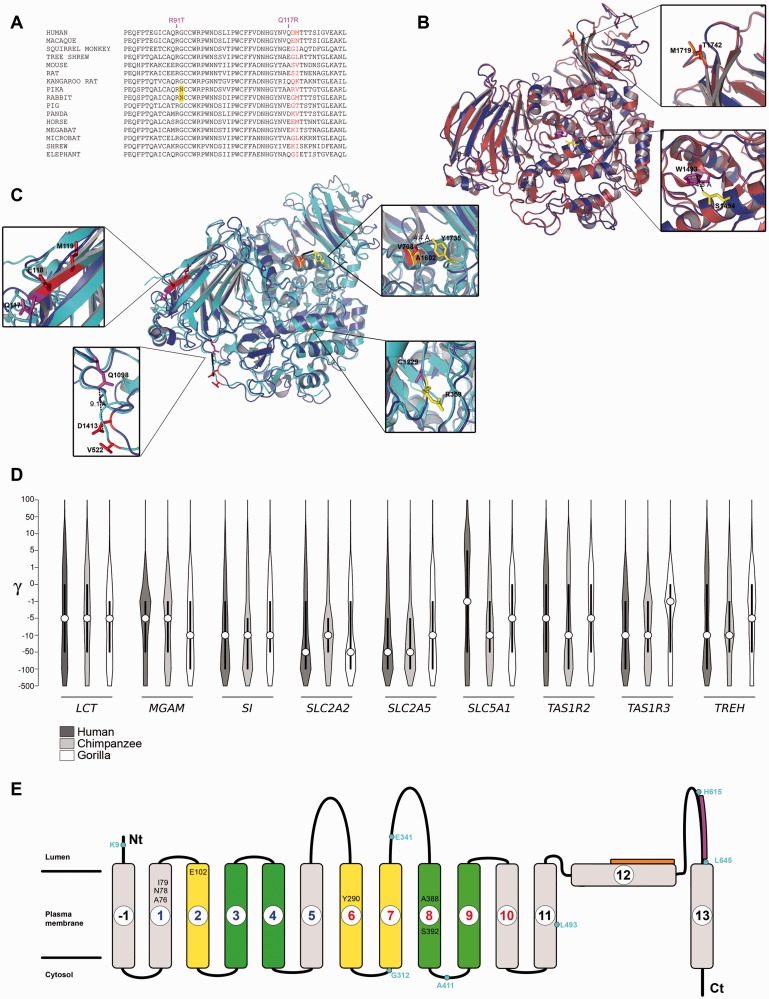
Parallel evolution at MGAM and SI, and lineage-specific selection. (*A*) Multiple alignment of SI amino acids 78–130 for a few of representative mammalian species. The location of mutations R91T and Q117R is shown. (*B* and *C*) Superimposition of the structure of the sucrase domain (SI, Protein Model Portal code: P14410 Model 2, blue) with glucoamylase (MGAM, PDB code: 3TON, red) (*B*) and with isomaltase (SI, PDB code: 3LPP, pale blue) (*C*). Enlargements highlight positively selected sites or residues subjected to pathological mutation located in the corresponding regions of the two different domains. Color codes are as in figure 2*A–C*. Human missense mutations affecting the protein sorting are reported in magenta. (*D*) Violin plot of selection coefficients (median, white dot; interquartile range, black bar). Selection coefficients (γ) are classified as strongly beneficial (100, 50), moderately beneficial (10, 5), weakly beneficial (1), neutral (0), weakly deleterious (−1), moderately deleterious (−5, −10), strongly deleterious (−50, −100), and inviable (−500). (*E*) Topological representation of SLC5A1; transmembrane helices forming the sugar- and hush-bundle are represented in yellow and green, respectively. The location of the stereo-specific and nonstereo-specific binding motifs is shown in magenta and orange, respectively. Positively selected sites in the human lineage are in cyan. Residues in black are involved in sugar or Na^+^ binding.

Although four glycosyl-hydrolase domains of SI and MGAM share limited sequence identity, their 3D structure is remarkably similar. Structural superimposition indicated that, in addition to the trefoil domain, other corresponding regions were targeted by selection (fig. 3*B* and *C*).

In SI, missense mutations responsible for congenital SI deficiency (CSID) or identified in chronic lymphocytic leukemia patients (CLL) have been shown to alter the cellular trafficking of the protein, its folding, membrane turnover and localization (Spodsberg et al. 2001; Rodriguez et al. 2013). We noted that mutations R91T (CLL, endoplasmic reticulum accumulation) and Q117R (CSID, missorting to the basolateral membrane) (Spodsberg et al. 2001; Rodriguez et al. 2013) immediately flank positively selected sites (fig. 3*A*). Three dimensional mapping and structural comparisons indicated that CSID mutations Q1098P, C1229Y, and W1493C (Propsting et al. 2003; Alfalah et al. 2009; Rodriguez et al. 2013) are located in close spatial proximity to positively selected sites in either SI or MGAM (fig. 3*B* and *C*).

### Parallel and Divergent Evolution of Brush-Border Proteins in Humans, Chimpanzees, and Gorillas

We next applied a population genetics-phylogenetics approach to study the evolution of brush-border genes in the human, chimpanzee, and gorilla lineages. Specifically, we used gammaMap (Wilson et al. 2011) that jointly uses intraspecific variation and interspecific diversity to estimate the distribution of selection coefficients (γ) along coding regions. gammaMap envisages 12 classes of γ, ranging from strongly beneficial (γ = 100) to inviable (γ = −500), with γ equal to 0 indicating neutrality.

We observed a general preponderance of codons evolving under negative selection (γ < 0) in all genes and in all species. The most striking difference was observed for *SLC5A1*, which showed a preponderance of negative γ values in chimpanzee and to a lesser extent in gorilla, but not in our species, where an appreciable fraction of codons showed γ values higher than 5 (fig. 3*D*). We thus used gammaMap to identify specific codons evolving under positive selection (cumulative probability > 0.80 of γ ≥ 1) (supplementary table S6, Supplementary Material online). Seven positively selected codons were identified for SLC5A1 in humans, none in chimpanzees or gorillas. Although two of these (A411 and H615) might have hitchhiked with a regulatory variant (see below), analysis of the remaining sites indicated that E341 and G312 flank one of the transmembrane helices composing the so-called “sugar bundle,” which forms extensive contacts with carbohydrate molecules (Sala-Rabanal et al. 2012) (fig. 3*E*). One additional site (L645) is in the immediate vicinity of a C-terminal luminal region that acts as a stereo-specific sugar binding region (fig. 3*E*) (Wimmer et al. 2009).

The location relatives to 3D structures of other positively selected sites (supplementary table S6, Supplementary Material online) are shown in figures 1*B*, 3*B*, 3*C*, and supplementary figure S4, Supplementary Material online.

### Preagricultural Origin of Most Positively Selected Alleles

We finally investigated whether natural selection acted on genes involved in carbohydrate digestion/absorption during the recent evolutionary history of human populations. We excluded *LCT* from this analysis, as its selection pattern has been described in detail (Tishkoff et al. 2007). Natural selection leaves signatures that can be detected using appropriate tests. For instance, the increase in frequency of a selected haplotype (selective sweep) may result in a temporary reduction in the level of genetic variability (measured by θ_W_ [Watterson 1975] and π [Nei and Li 1979]) and in a shift of the site frequency spectrum, leading to a deficiency of intermediate frequency alleles (indicated by negative values of Tajima’s *D* [Tajima 1989]). Also, a selective sweep may determine an excess of high frequency derived alleles (which can be assessed with the normalized Fay and Wu’s *H* [DH] test [Zeng et al. 2006]) and low nucleotide diversity associated with the derived allele (Barreiro et al. 2009). This latter feature can be searched for using the DIND test (Barreiro et al. 2009). Thus, using the 1000 Genomes Pilot Project data (1000 Genomes Project Consortium et al. 2010) for YRI, CEU, and CHBJPT, we estimated nucleotide diversity and Tajima’s *D* (Tajima 1989) over whole gene regions. We also calculated pairwise *F*_ST_, an estimate of population genetic differentiation, and performed the DIND test for all SNPs mapping to these genes and in their 50 kb flanks (25 kb up- and down-stream). For all tests statistical significance (in terms of percentile rank) was obtained by deriving empirical distributions*;* coalescent simulations were also performed for the DIND test*.* We considered genes as positive selection targets if significant results were obtained for the same population in at least two statistics based on different features; we also considered SNPs with a significant DIND test in all populations or with extremely high DIND ranks (>0.999). Moreover, we obtained normalized values for Fay and Wu’s H (DH) (Zeng et al. 2006), in sliding windows along the analyzed genomic regions; DH was used as a confirmatory signature but not in the initial detection of selection targets (supplementary table S1, Supplementary Material online).

**Fig. 4.—:**
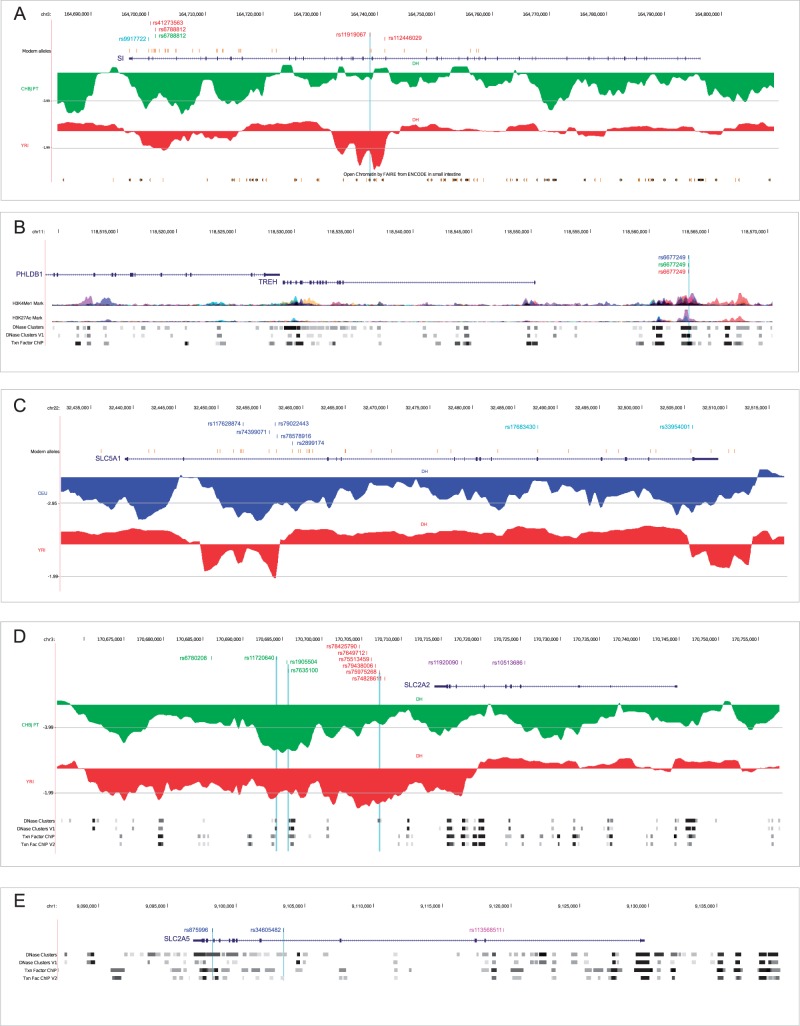
Location of the most likely selection targets. Candidate targets in human populations and their genomic locations (GRCh37/hg19) are shown for *SI* (*A*), *TREH* (*B*), *SLC5A1* (*C*), *SLC2A2* (*D*), and *SLC2A5* (*E*) within the UCSC Genome Browser view. Relevant ENCODE annotation tracks are shown as gray horizontal shading or colored peaks in case of histone marks. Candidate selection targets falling in putative regulatory regions are indicated with cyan vertical lines. For *SI*, *SLC5A1*, and *SLC2A2* a sliding-window analysis of DH is also shown in green (YRI), red (CHBJPT), or blue (CEU). The gray horizontal line represents the fifth percentile of DH. Variants in blue, red and green represent selection targets in CEU, CHBJPT, and YRI, respectively. The location of variants cataloged as modern-human-specific sites are shown in orange. Additional color codes are as follows: cyan, positively selected sites in the human lineage detected by gammaMap; violet, GWAS SNPs; magenta, eQTL.

**Fig. 5.—:**
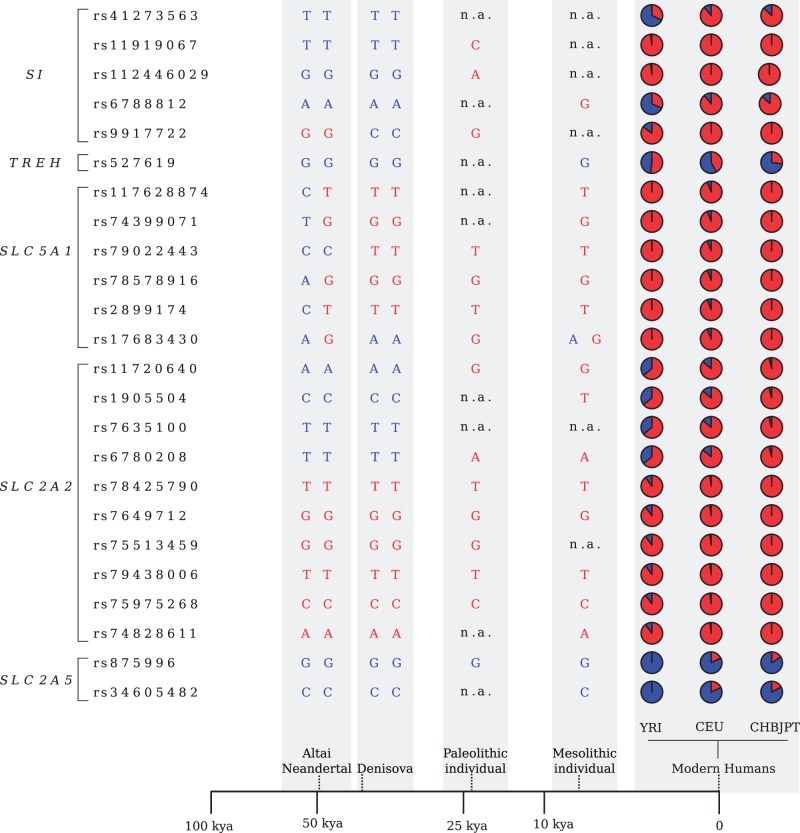
Positively selected variants in human populations. Genotype data are shown for a Neandertal, a Denisova, an Upper Paleolithic Siberian, and a Mesolithic hunter-gatherer; allele frequencies are shown for modern human populations (pie-charts). Only one allele is reported when coverage was not sufficient for genotype inference. Blue and red colors indicate ancestral and derived alleles, respectively. A temporal line with the approximate ages of the individuals is also reported (kya: thousands of years ago).

In *SI*, the DIND test detected two outlier linked variants in YRI, which also had unusually high *F*_ST_ (table 2); rs6788812 represented a DIND outlier in CHBJPT, as well, and was located in a local valley of DH for this population (in line with DH having maximum power for high-frequency sweeps [Zeng et al. 2006]) (fig. 4*A* and table 2). These results suggest that a common selective sweep determined the frequency increase of these variants in Asia and Africa. No selection signal was detected in CEU and analysis of ancient DNA samples indicated that the Denisova and Altai Neandertal (Meyer et al. 2012; Prufer et al. 2014) carry the ancestral allele, whereas a Mesolithic European individual from the La Brana-Arintero site (Olalde et al. 2014) harbors the derived allele at rs6788812 (table 2 and fig. 5).

**Table 2:** Candidate Targets of Positive Selection in Human Populations

Gene	SNP ID	Derived Allele^a^	DAF^b^			DIND Rank (population)	DIND *P* Value^c^ (population)	*F*_ST_ Rank (comparison)	Notes
			**YRI**	**CEU**	**CHBJPT**				
***SI***	rs41273563	C	0.32	0.89	0.87	0.97 (YRI)	0.031 (YRI)	0.96 (YRI/CHBJPT)	
	rs11919067	C	0.98	1	1	>0.999 (YRI)	<0.001 (YRI)	—	Modern-human-specific site
	rs112446029	A	0.98	1	1	0.99 (YRI)	0.008 (YRI)	—	Modern-human-specific site
	rs6788812	G	0.32	0.89	0.87	0.98 (YRI) 0.98 (CHBJPT)	0.024 (YRI) 0.044 (CHBJPT)	0.95 (YRI/CEU)	
	rs9917722	G	0.85	1	1	—		—	Modern-human-specific site; identified by gammaMap
***TREH***	rs527619	A	0.52	0.42	0.27	0.98 (YRI), 0.97 (CEU), 0.99 (CHBJPT)	0.007 (YRI), 0.073 (CEU), 0.081 (CHBJPT)	—	
***SLC5A1***	rs117628874	T	1	0.94	1	0.95 (CEU)	0.012 (CEU)	—	Modern-human-specific site
	rs74399071	G	1	0.94	1	0.96 (CEU)	0.012 (CEU)	—	Modern-human-specific site
	rs79022443	T	1	0.94	1	0.97 (CEU)	0.010 (CEU)	—	
	rs78578916	G	1	0.94	1	0.98 (CEU)	0.002 (CEU)	—	Modern-human-specific site
	rs2899174	T	1	0.94	1	0.95 (CEU)	0.012 (CEU)	—	Modern-human-specific site
	rs17683430	G	1	0.94	1	—		—	Modern-human-specific site; identified by gammaMap
***SLC2A2***	rs11720640	G	0.64	0.86	0.96	0.99 (CHBJPT)	0.047 (CHBJPT)	0.95 (CHBJPT/YRI)	In LD with rs11920090 and rs10513686 (GWAS)
	rs1905504	T	0.64	0.86	0.96	>0.999 (CHBJPT)	0.047 (CHBJPT)	0.95 (CHBJPT/YRI)	In LD with rs11920090 and rs10513686 (GWAS)
	rs7635100	G	0.64	0.86	0.96	>0.999 (CHBJPT)	0.047 (CHBJPT)	0.95 (CHBJPT/YRI)	In LD with rs11920090 and rs10513686 (GWAS)
	rs6780208	A	0.64	0.86	0.96	>0.999 (CHBJPT)	0.047 (CHBJPT)	0.95 (CHBJPT/YRI)	In LD with rs11920090 and rs10513686 (GWAS)
	rs78425790	T	0.90	0.98	1	0.97 (YRI)	0.028 (CHBJPT)	0.99 (YRI/CEU), 0.99 (YRI/CHBJPT)	
	rs7649712	G	0.90	0.98	1	0.98 (YRI)	0.026 (YRI)	0.99 (YRI/CEU), 0.99 (YRI/CHBJPT)	
	rs75513459	G	0.90	0.98	1	0.97 (YRI)	0.031 (YRI)	0.99 (YRI/CEU), 0.99 (YRI/CHBJPT)	
	rs79438006	T	0.90	0.98	1	0.96 (YRI)	0.034 (YRI)	0.99 (YRI/CEU), 0.99 (YRI/CHBJPT)	
	rs75975268	C	0.90	0.98	1	0.97 (YRI)	0.030 (YRI)	0.99 (YRI/CEU), 0.99 (YRI/CHBJPT)	
	rs74828611	A	0.90	0.98	1	0.98 (YRI)	0.029 (YRI)	0.99 (YRI/CEU), 0.99 (YRI/CHBJPT)	
***SLC2A5***	rs875996	A	0	0.18	0.16	0.97 (CEU)	0.014 (CEU)	0.99 (YRI/CEU)	In LD with rs113568511 (eQTL)
	rs34605482	T	0	0.18	0.17	0.95 (CEU)	0.034 (CEU)	0.99 (YRI/CEU)	In LD with rs113568511 (eQTL)

^a^To avoid misattribution (Hernandez et al. 2007), the derived allele was inferred by parsimony through incorporating sequence information for at least four primate species.

^b^DAF.

^c^*P* value calculated by coalescent simulations.

In YRI another variant (rs11919067) had an extremely high DIND rank and a linked SNP (rs112446029) represented a DIND outlier, although with lower rank (table 2); both variants have high DAF in YRI, whereas the derived allele is fixed outside Africa (table 2). Sliding-window analysis of DH in YRI detected a local valley where rs11919067 is located (fig. 4*A*). Overall, these results suggest that a selective sweep drove the frequency increase of these variants in all populations and that the process is complete in non-Africans. Interestingly, rs11919067 and rs112446029 have been cataloged in a list of ‘modern-human-specific sites’—that is, positions where the Denisova or Altai Neandertal sequences display the ancestral allele, whereas most modern humans carry the derived allele (Prufer et al. 2014) (table 2 and fig. 5). The catalog also includes rs9917722 (T1802S), which we identified in the gammaMap analysis (table 2, supplementary table S6, Supplementary Material online). Analysis of all modern-human-specific sites in *SI* (fig. 4*A*) indicated that they mainly cluster in two regions, one where rs9917722 and rs6788812 are located, and the other encompassing rs11919067 and rs112446029. In YRI rs9917722 shows no linkage disequilibrium (LD) with rs11919067 and rs6788812 (*r*^2 ^= 0.003 and 0.085, respectively). Overall, these data suggest that distinct selective events have occurred at SI after the modern-human lineage split from the common ancestor with Denisovans and Neandertals. Interestingly, analysis of an Upper Paleolithic sample from Siberia (Raghavan et al. 2014) indicated that this individual carried the derived allele at rs11919067, rs112446029, and rs9917722 (fig. 5).

Signals of positive selection in all populations were also detected at another brush-border enzyme, TREH. Indeed, the same *TREH* variant (rs527619) was identified as a DIND outlier in all three populations, although with different DAF (table 2 and fig. 4*B*).

As for transporters, *SLC5A1* showed reduced nucleotide diversity in CHBJPT and low Tajima’s *D* in CEU and CHBJPT (supplementary table S7, Supplementary Material online). The DIND test detected five linked outlier variants in CEU with a DAF of 0.94; the derived allele is fixed in YRI and CHBJPT (table 2). The SNPs are in a local valley of DH in CEU, and a very local and limited reduction in DH was also observed in YRI (DH loses power at sweep completion) (fig. 4*C*). Four of the five *SLC5A1* SNPs we detected are listed in the modern-human-specific site catalog, which also includes rs17683430 (A411T, detected by gammaMap) (table 2, supplementary table S6, Supplementary Material online). Analysis of modern-human-specific sites along the *SLC5A1* gene indicated that they are scattered across a relatively large region with a clustering around the five variants detected (fig. 4*C*); in CEU these are in tight LD with rs17683430 and with rs33954001 (also detected by gammaMap, supplementary table S6, Supplementary Material online) (*r*^2 ^> 0.86), suggesting these SNPs hitchhiked to high frequency due to LD with one of the DIND outlier variants. Analysis of the Mesolithic and Paleolithic samples (Olalde et al. 2014; Raghavan et al. 2014) revealed that the derived allele was already present at all selected variants (fig. 5).

*SLC2A2* also showed low Tajima’s *D* values in CHBJPT (supplementary table S7, Supplementary Material online). Four variants were DIND outliers in CHBJPT and displayed unusually high *F*_ST_ in the YRI/CHBJPT comparison. The variants have high DAF in CHBJPT (table 2) and are located in a local DH valley, strongly supporting selective sweep has occurred in Asian populations (fig. 4*D*). Interestingly, in CEU the four variants are in tight LD (*r*^2 ^> 0.9) with two GWAS SNPs (rs11920090 and rs10513686) associated with fasting glucose-related traits and gamma-glutamyl transferase (GGT) levels (Dupuis et al. 2010; Chambers et al. 2011; Manning et al. 2012). Both the Mesolithic and the Paleolithic samples carried the derived allele at most SNPs (fig. 5). Thus, in analogy to the *SLC5A1* and *SI* variants, the selected haplotype was present in the Paleolithic (fig. 5).

In *SLC2A2*, the DIND test also detected six outliers in YRI, which also display high *F*_ST_ values (table 2). These variants have a DAF of 0.90 in YRI and fall in a DH valley (fig. 4*D*); the derived allele is fixed or almost fixed in non-Africans, suggesting a complete sweep that predated the split of modern humans from Neandertals and Denisovans, as these hominins also carry the derived alleles (fig. 5).

Finally, in *SLC2A5* two DIND outlier variants in CEU also displayed a high *F*_ST_ ranks (table 2), suggesting that a selective sweep has occurred in CEU. The two variants are in LD (*r*^2 ^= 0.76) with rs113568511, identified as an eQTL (expression quantitative trait locus) for *SLC2A5* in lymphoblastoid cell lines (Lappalainen et al. 2013).

Several selected variants we detected map within ENCODE functional elements (fig. 4).

Overall, we analyzed eight genes (*LCT* was omitted) and we found one with a significant DIND test for the same variant in three populations (*TREH*) and three with at least two variants showing outlier values both for the DIND and *F*_ST_ tests (*SLC2A2*, *SI*, and *SLC2A5*) (table 2). To obtain an estimate of whether these findings are unusual and of the incidence of false positives, we adopted a resampling approach. Specifically, we drew 100 samples of eight randomly selected genes and we calculated the DIND tests and *F*_ST_ for all variants mapping to these genes. For each sample we counted the number of positively selected genes, defined as those carrying at least one variant with significant DIND test in three populations or at least two variants showing outlier values both for the DIND and *F*_ST_ tests in the same population. Results indicated that the probability of drawing a set of genes showing the same or a higher number of selected genes as those in the brush-border set is 0.02.

## Discussion

### Adaptive Evolution in Mammals

We explored the evolutionary history of genes encoding brush-border proteins involved in carbohydrate digestion and absorption. This decision was based on the well-accepted concept that the availability of food resources is a driver of pivotal importance in evolution in mammals and that individual mammalian lineages might have adapted to specialized diets (e.g., insects, crustaceans) or lifestyles (e.g., flight).

We found evidence of positive selection at the four brush-border enzymes, indicating stronger selective pressure compared with transporters and taste receptors. Episodic positive selection was also detected for several mammalian lineages. Although for *TREH* two positively selected lineages (microbat and platypus) have a diet that includes trehalose, mammals showing evidence of positive selection at *MGAM* and *SI* display different food habits. Thus, as previously reported for *TAS1R2* (Zhao et al. 2010; Jiang et al. 2012), inference of the underlying selective pressures remains uncertain. Nonetheless, we detected positive selection at *SI* in both bat species (megabat is frugivorous, microbat insectivore), with microbat also showing selection signatures at *MGAM* and *TREH*. As an adaptation to flight, bats generally display a reduced small intestinal nominal surface area compared with nonflying mammals, and resort to higher sugar paracellular absorption as a compensation (Caviedes-Vidal et al. 2007). Because polysaccharides require digestion before they can be metabolized, fast and efficient digestion of complex sugars would be strongly advantageous in these species, which daily ingest large amounts of food (up to 50% of their body weight) to meet energy requirements. Whether positive selection at *SI* and *MGAM* is part of a more general adaptation to flight in these animals remains an interesting possibility worth further investigation.

### Positively Selected Sites in Enzyme Encoding Genes

The rate of starch-generated glucose depends on the activity of MGAM and SI, which have complementary substrate specificity in humans (Sim et al. 2010). In a few instances we found the corresponding residues of MGAM and SI to be targeted by selection, indicating an important role for these sites. An interesting possibility is that some selected sites in SI and MGAM evolved to hone the folding, cellular trafficking, and membrane turnover of these enzymes, depending on specific molecular (e.g., interaction with chaperones) or physiological (e.g., body temperature) features of distinct mammals. In fact, some of the identified selected sites are located in close spatial proximity to SI missense mutations that affect the enzyme's posttranslational fate, sometimes showing temperature-sensitive effects (Propsting et al. 2003; Alfalah et al. 2009; Rodriguez et al. 2013). In analogy, an LCT missense mutation associated with congenital lactase deficiency (G1363S), has been shown to alter protein trafficking and folding, partially depending on temperature (Behrendt et al. 2009).

### Adaptive Events in Primates and Human Populations

Our study was also motivated by the observation that one of the most important turning-points of human history, the introduction of agriculture, resulted in a dietary shift in terms of carbohydrate intake. In this respect, the availability of genetic information for other primates and for preagricultural human populations allows the opportunity to address the tempo and mode of evolution for genes involved in carbohydrate digestion and absorption.

A notable observation is the different evolutionary fate of *SLC5A1* in humans versus chimpanzees and gorillas. Still, we note that, whereas some sites positively selected in the human *SLC5A1* gene are likely involved in sugar binding, the signal we detected is partially accounted for by hitchhiking of coding variants with the intronic positive selection target(s), as population genetic analysis indicated.

Integration of different tests can improve the power to detect selective sweeps and, importantly, allows identification of the causal variant(s) (Grossman et al. 2013). Our approach includes the DIND test, which is powerful in most DAF ranges (Barreiro et al. 2009; Fagny et al. 2014) and less sensitive than iHS (Integrated Haplotype Score) to low genotype quality or low coverage (i.e., it is well suited for the 1000G data) (Fagny et al. 2014). DIND results were combined with pairwise *F*_ST_ analyses and nucleotide diversity or Tajima’s *D*, whereas DH (Zeng et al. 2006) was calculated in sliding-windows to account for local events and, for this reason, used as an a posteriori validation. These analyses indicated that five out of the nine genes we analyzed have been targeted by selection during the history of human populations, with *SI* and *SLC2A2* having experienced distinct events targeting different variants. The majority of sweeps we detected occurred in all analyzed populations, although in some instances they have reached completion (e.g., *SI* and *SLC2A2* in non-Africans and *SLC5A1* in non-Europeans) or proceeded with different timing/strength (e.g., *TREH*).

The availability of an increasing number of ancient DNA sequences allows the unprecedented opportunity to define the time in human history when selection operated, in turn providing information on the possible selective pressures. Based on the sequencing of a Denisova and a Neandertal individual, and on allele frequency in extant human populations, Prufer et al. (2014) compiled a list of modern-human-specific-alleles, suggested to represent changes that were most important during the recent evolutionary history of our species. Results herein indicate that modern alleles at *SLC5A1* and *SI* were indeed driven to high frequency by natural selection in human populations. Nevertheless, most of these positively selected modern alleles were already present in the Mesolithic and Paleolithic and, therefore, predate the emergence of agriculture. Whether the onset of selection occurred before the Paleolithic or these alleles segregated as neutral standing variation in these early populations remains to be evaluated, possibly through the sequencing of additional ancient samples. Although with uncertainty due to possible gene conversions, the initial expansion of the *AMY1* copy number was dated around 200,000 years ago, a time frame that might coincide with the introduction of starch-rich underground storage organs (USOs) as food sources in hominin diet (Perry et al. 2007). USOs are thought to have played an important role in human evolution (Laden and Wrangham 2005). Thus, agriculture might have spurred the frequency increase of variants that were already weakly adaptive in hunter-gatherers, resulting in a continuum rather than an abrupt onset of selective events. A similar concept has been proposed for traits unrelated to diet (Olalde et al. 2014).

As for the more recent selective event at *SLC2A5*, it is worth noting that some degree of fructose intolerance is widespread in humans, and fructose absorption is increased by the coingestion of glucose and is reduced by the presence of sorbitol (Skoog and Bharucha 2004). Thus, selection at *SLC2A5* might have been driven by the domestication in temperate areas of fruit crops (e.g., apples and pears) that contain excess fructose plus sorbitol (Skoog and Bharucha 2004). Clearly, it would be extremely interesting to test whether the positively select variant identified herein (and which is in LD with an eQTL), modulates fructose absorptive capacity.

### Selection Targets in Regulatory Regions

In analogy to the well-known selection targets at the *LCT* locus (Tishkoff et al. 2007), the selection signatures we identified in human populations target noncoding polymorphisms, supporting the view that most adaptive changes affect regulatory elements (Grossman et al. 2013). We suggest that regulatory variants may also represent the selection target at the dog *MGAM* and *SLC5A1* genes. Although the analyses we performed were not specifically devised to search for recent selective events in dogs, and surely lack power in this respect, the candidate coding variants Axelsson et al. (Axelsson et al. 2013) proposed can be analyzed within the framework of the known mammalian phylogeny. Overall, these analyses suggest that coding variants are not likely selection targets in the canine *MGAM* and *SLC5A1* genes, in line with the observation that the expression of *MGAM* is higher in dogs compared with wolves (Axelsson et al. 2013).

Deeper understanding of the evolutionary processes associated with human dietary shifts is expected to provide valuable information concerning the susceptibility of human populations to metabolic diseases.

Data herein indicate that the selection targets at *SLC2A2* are in phase with the risk allele for fasting glucose levels and with the nonrisk allele for GGT levels. This opens the question as to whether the disease alleles hitchhiked with the selected variant, or might be accounted for by the selected haplotype. In either case, further analyses will be required to determine which phenotype selection acted upon.

## Supplementary Material

Supplementary figures S1–S4 and tables S1–S7 are available at *Genome Biology and Evolution* online (http://www.gbe.oxfordjournals.org/).

Supplementary Data
