# Supplementary material for: Natural Selection at the Brush-Border: Adaptations to Carbohydrate Diets in Humans and Other Mammals
Source: Genome Biol Evol. 2015 Sep 11;7(9):2569–84. doi: 10.1093/gbe/evv166 (PMC4607523; doi:10.1093/gbe/evv166)
Supplement: Supplementary Data [file supp_7_9_2569__index.html]

Natural selection at the brush-border: adaptations to carbohydrate diets in humans and other mammals — Natural Selection at the Brush-Border: Adaptations to Carbohydrate Diets in Humans and Other Mammals — Supplementary Data 

# Natural Selection at the Brush-Border: Adaptations to Carbohydrate Diets in Humans and Other Mammals

## Supplementary Data

files

- Supplementary Data - pdf file
