# Supplementary material for: Natural Selection at the Brush-Border: Adaptations to Carbohydrate Diets in Humans and Other Mammals
Source: Genome Biol Evol. 2015 Sep 11;7(9):2569–84. doi: 10.1093/gbe/evv166 (PMC4607523; doi:10.1093/gbe/evv166)
Supplement: Supplementary Data [file supp_evv166_Attached_file_3a_Pontremoli-Supplemental-data.pdf]

Supplementary Figure S1. Branch-site analysis of positive selection for *LCT*, *SLC2A2*, and *TREH*.

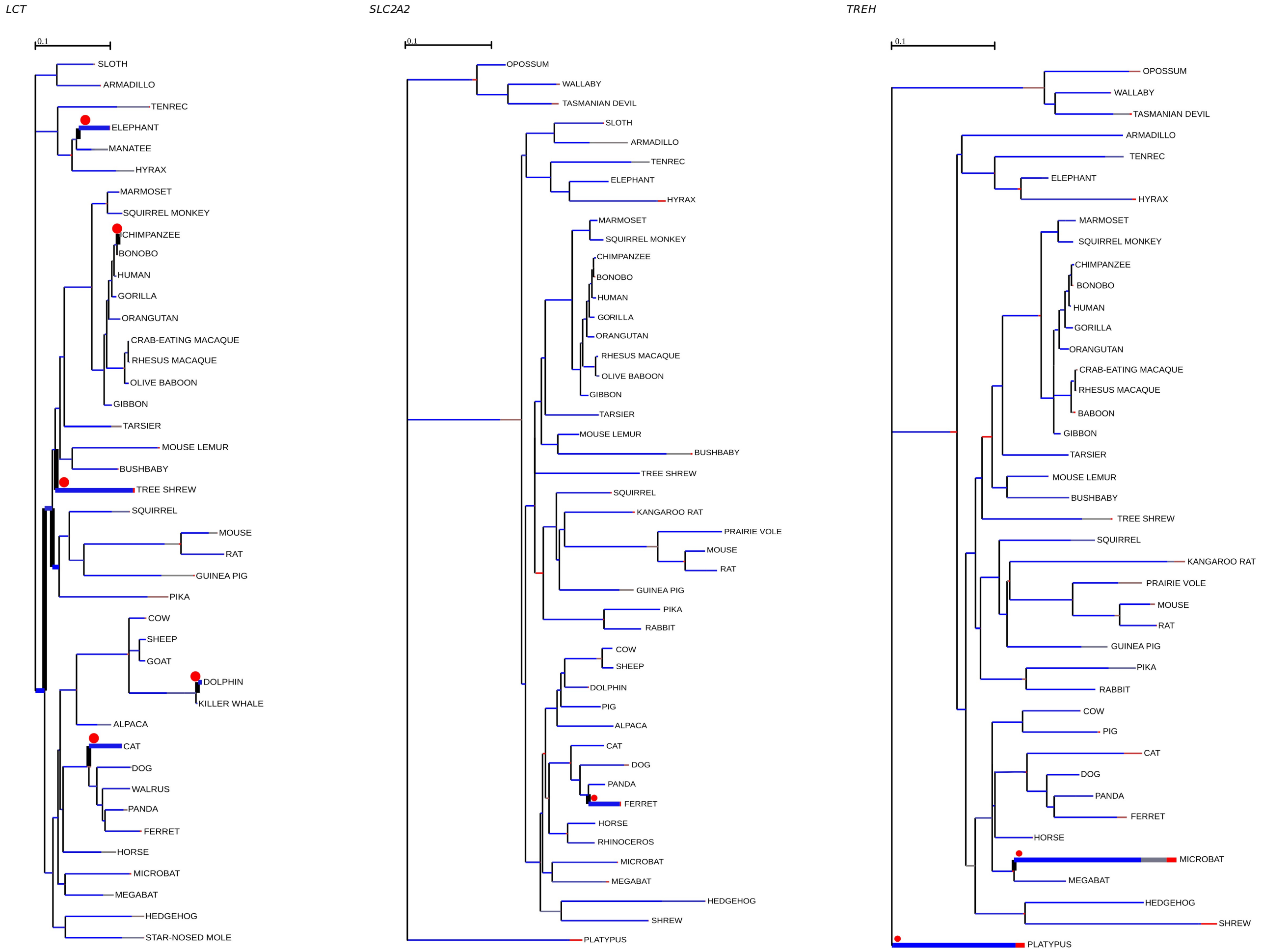

Supplementary Figure S2. Branch-site analysis of positive selection for *MGAM* and *SI*.

MGAM region 1

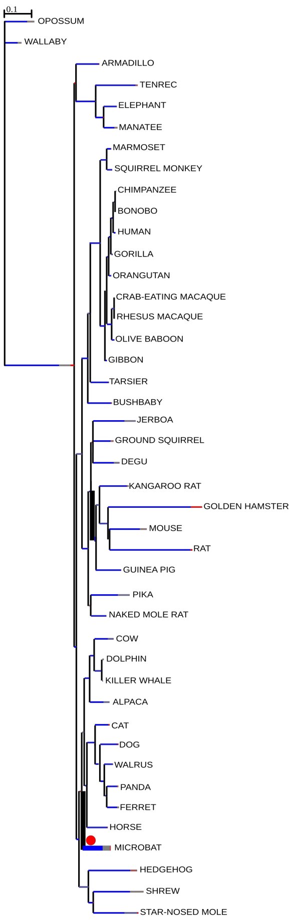

MGAM region2

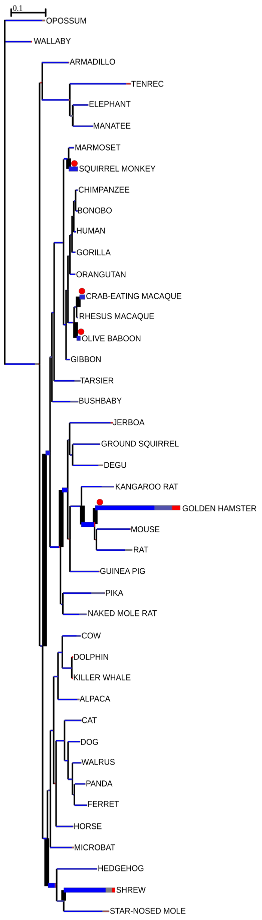

SI region 1

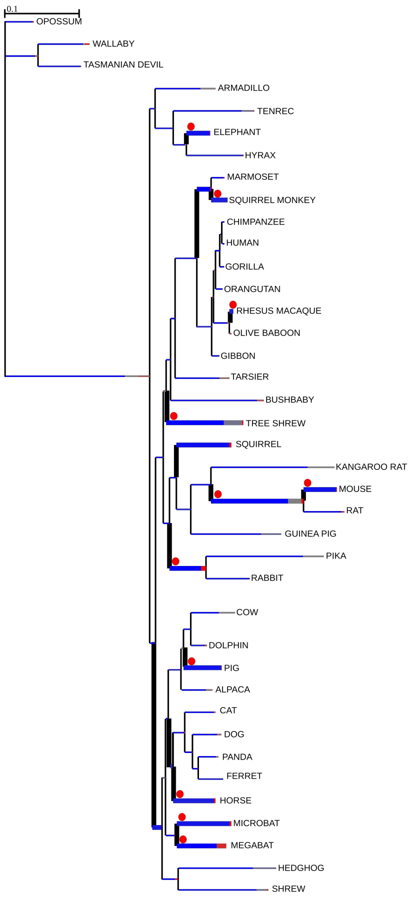

SI region 2

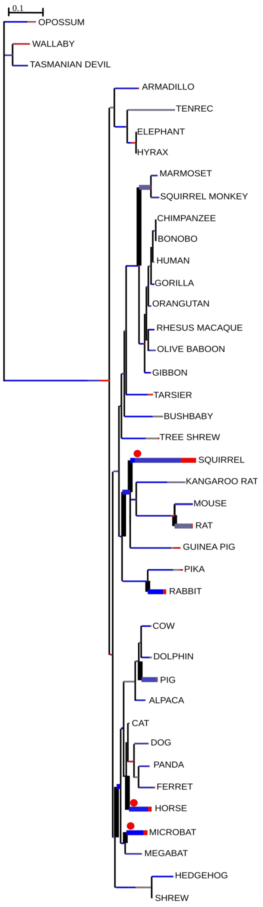

Supplementary Figure S3. Recombination rate and GC content for brush-border genes.

A

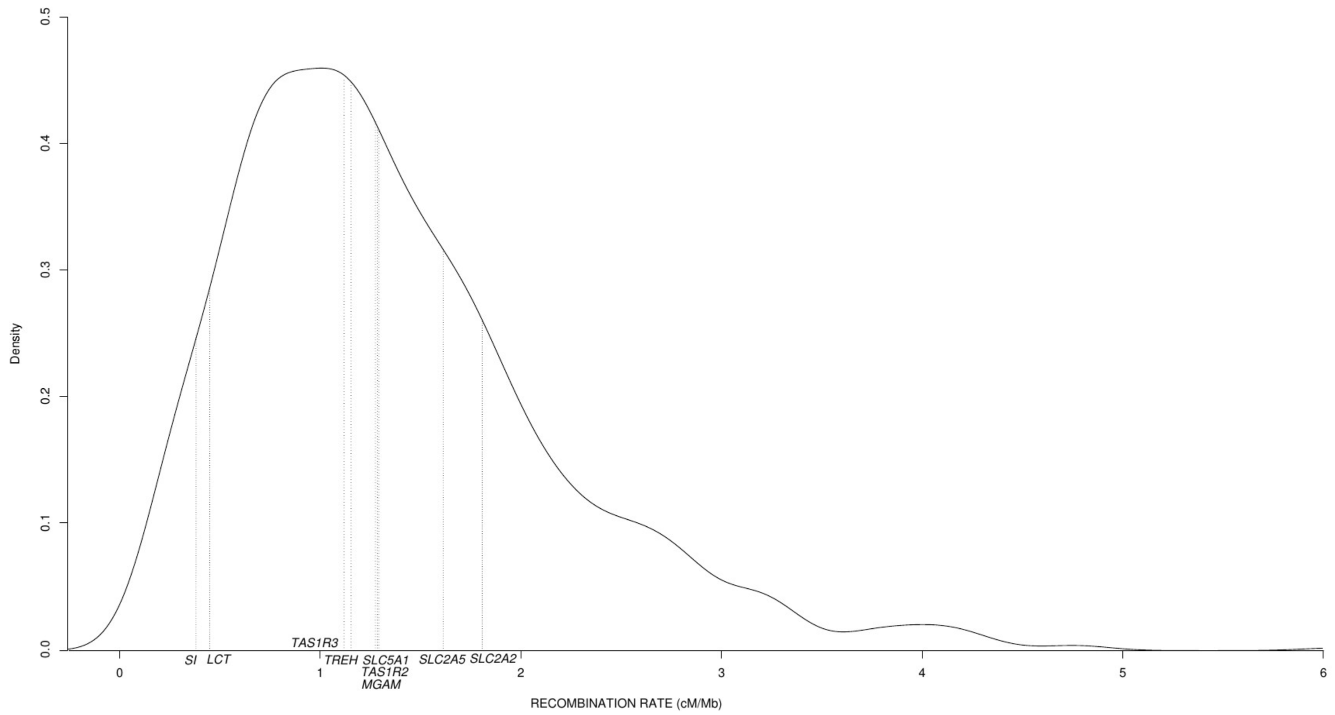

B

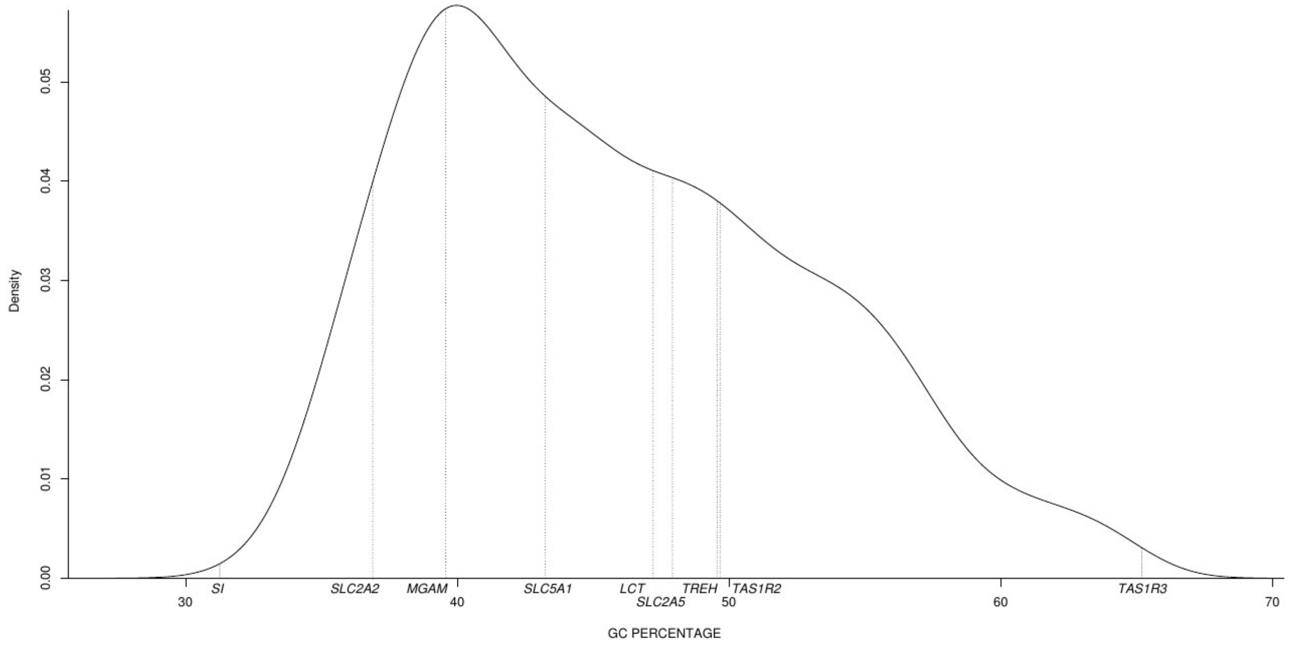

Supplementary Figure S4. Analysis of positively selected sites in MGAM and SI.

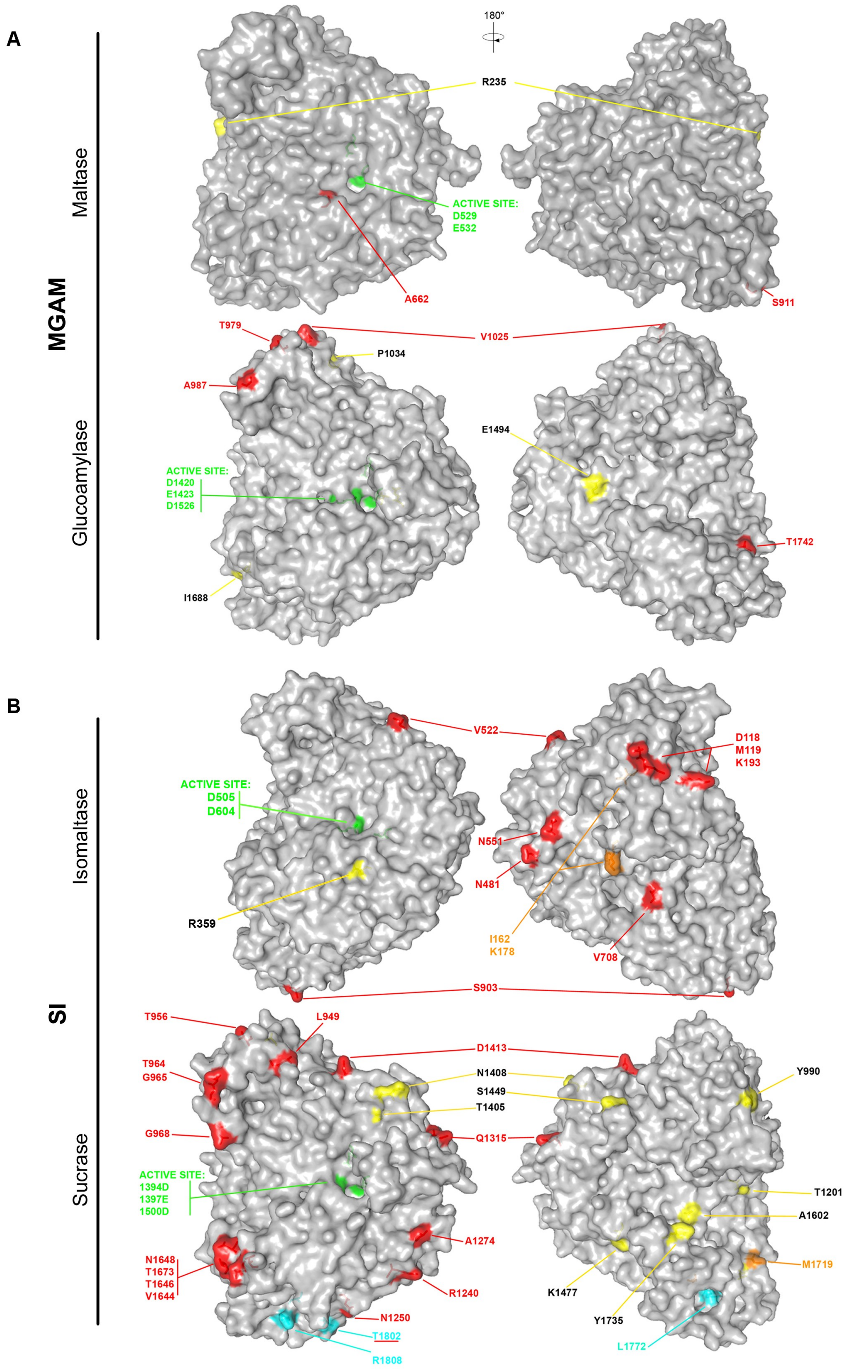

Supplemental Figure Legends

Supplementary Figure S1. Branch-site analysis of positive selection for *LCT*, *SLC2A2*, and *TREH*. BS-REL analysis for the three genes. Branch lengths are scaled to the expected number of substitutions per nucleotide, and branch colors indicate the strength of selection (dN/dS). Red, positive selection (dN/dS>5); blue, purifying selection (dN/dS = 0); grey, neutral evolution (dN/dS = 1). The proportion of each color represents the fraction of the sequence undergoing the corresponding class of selection. Thick branches indicate statistical support for evolution under episodic diversifying selection as determined by BS-REL. Red dots denote branches that were confirmed to be under positive selection using the *codeml* branch-site models (after FDR correction for multiple tests). When two sequences display 100% identity, one of them is removed by BS-REL. Thus, in very limited number of instances species in trees may display fewer species than in Table 1.

Supplementary Figure S2. Branch-site analysis of positive selection for *MGAM* and *SI*. Analyses were performed separately for the two regions, according to the location of recombination breakpoints. Colors and explanations as in Figure S1.

Supplementary Figure S3. Recombination rate and GC content for brush-border genes. (A) Distribution of recombination rates for the ~1000 genes used to generate the control distribution. The recombination rate for the 9 brush-border genes is shown. (B) the same as (A) for GC content.

Supplementary Figure S4. Analysis of positively selected sites in MGAM and SI. (A) Surface representation of MGAM (maltase domain, PDB code: 3L4V; glucoamylase domain, PDB code: 3TON). (B) Surface representation of SI (isomaltase domain, PDB code: 3LPP; sucrase domain, Protein Model Portal code: P14410, Model 2). In (A) and (B) color codes as follows: red, positively selected sites in the whole phylogeny; yellow, lineage-specific sites; orange, positively selected sites in the chimpanzee lineage; cyan, positively selected sites in the human lineage; green, catalytic residues.

Supplementary Table S1. Summary of algorithms, programs, and tests used for bioinformatics analysis.

Computational resources/ statistics	Description	Cutoff used	References
Evolutionary analysis inter-species			
**Databases**			
NCB I (National Center for Biotechnology Information) database	The National Center for Biotechnology Information database provides access to biomedical and genomic information.		
**Server**			
DataMonkey sever	Web server for HyPhy, a computational phylogenetics software package developed to perform maximum likelihood analyses of genetic sequence data and equipped with tools to test various statistical hypotheses.		(Delport et al. 2010)
**Utilities**			
EnsemblCompara GeneTrees	The database allows performing cross-species analyses to infer gene orthology and paralogy by using phylogenic gene trees generated by maximum likelihood.		(Vilella et al. 2009)
RevTrans 2.0 utility	It virtually translates a set of DNA sequences, aligns the peptide sequences, and uses this as a scaffold to construct the corresponding DNA multiple alignment.		(Wernersson and Pedersen 2003)
TrimAl	This tool allows the automated removal of spurious sequences or poorly aligned regions from a multiple sequence alignment.		(Capella-Gutierrez et al. 2009)
GARD (Genetic Algorithm Recombination Detection)	It is a genetic algorithm of the Hyphy package developed to search for recombination breakpoints in multiple sequence alignments and to identify putative recombinant sequences.	<0.01	(Kosakovsky Pond et al. 2006)
SLAC (Single Likelihood Ancestor Counting)	A tool from the Hyphy package for the estimation of the average dN/dS ratio; it uses likelihood-based branch lengths, nucleotide and codon substitution parameters and ancestral sequence reconstructions.		(Kosakovsky Pond and Frost 2005)
PhyML	This software estimates maximum likelihood phylogenies from alignments of nucleotide or amino acid sequences.		(Guindon et al. 2009).
codeml	This software is from the PAML package and applies likelihood ratio tests to compare models of gene evolution that allow or disallow a class of codons to evolve with dN/dS >1.	≤0.05	(Yang 2007)
BEB (Bayes Empirical Bayes analysis)	This method is used to identify positively selected sites; it calculates the posterior probability that each codon is from the site class with	≥0.9	(Anisimova et al. 2002; Yang et al. 2005)

	dN/dS >1 (under models allowing dN/dS>1).		
MEME (Mixed Effects Model of Evolution)	This methods identifies positively selected sites by allowing the distribution of dN/dS to vary from site to site and from branch to branch at a site, thus detecting both pervasive and episodic positive selection. It is included in the HyPhy package.	≤0.1	(Murrell et al. 2012)
BSREL (Branch-site REL)	This tool performs a series of LRT tests to find lineages on which a proportion of sites evolve with dN/dS > 1, without making any a priori assumptions.	≤0.05	(Kosakovsky Pond et al. 2011)
Population genetics-phylogenetics analysis			
**Utilities**			
gammaMap	This program is based on a combined population genetics-phylogenetics model of selection. It estimates the distribution of selection coefficients, and allows localization of the signal of selection using a Bayesian sliding window approach. The signature of selection is detected from the contrast in the dN/dS ratio within and between species.	>0.8	(Wilson et al. 2011)
Population genetics analysis			
**Database**			
1000 Genomes	This database collects information about human genetic variation.		(1000 Genomes Project Consortium et al. 2010)
**Browser**			
UCSC table browser	Provides access to information about location and annotation of genomic regions.		(Karolchik et al. 2004)
**C++ libraries**			
GeCo++	This library allows to manage genomic element annotation, sequences, and positional genomic features; it provides users with tools to keep track of genomic variations.		(Cereda et al. 2011)
libsequence	This library facilitates writing and implementation of evolutionary genetics applications; it is mainly dedicated to the analysis of SNP data.		(Thornton 2003)
**Statistics**			
θ_W	This parameter estimates the expected per site heterozygosity.		(Watterson 1975)
π	Is defined as the average number of nucleotide differences per site between two DNA sequences.		(Nei and Li 1979)

Tajima's D	This test is based on the allele frequency spectrum (i.e. the distribution of allele frequencies at polymorphic sites); low negative values of D indicate an excess of rare alleles and suggest either purifying or positive selection.		(Tajima 1989)
DH	The test is based on the idea that directional selection at one site may drive linked mutations to high frequency; this also applies to derived alleles (which usually display lower frequency). Negative values indicate an excess of high frequency derived alleles and represent a signature of selective sweeps.	≤5th	(Fay and Wu 2000; Zeng et al. 2006)
F_{ST}	This parameter, also known as fixation index, measures variations in the allele frequency between two populations. F_{ST} largely depends on demographic history (which affects all loci equally) but natural selection may drive allele frequencies to differ more or less than expected on the basis of demography alone. Specifically, local adaptation may cause an allele to increase in frequency in one population and therefore result in high F_{ST} (high differentiation with another population)	≥95th	(Wright 1950)
DIND test (Derived Intra-allelic Nucleotide Diversity)	The DIND test evaluates haplotype homozygosity. It is based on the difference of nucleotide diversity between haplotypes carrying the derived and the ancestral alleles. It has higher power to detect recent selective events compared with the commonly used sequence-based neutrality tests.	≥95th	(Barreiro et al. 2009)
**Utilities**			
cosi package	This program performs coalescent simulation of sequence variation, considering human demographic history models		(Schaffner et al. 2005)
LiftOver tool	This tool, available from the UCSC genome browser, converts a given genome position from a genome assembly to the corresponding position in another assembly.		(Kuhn et al. 2013)

Supplementary Table S2. List of mammalian species.

Common name	Scientific name
Alpaca	*Vicugna pacos*
Armadillo	*Dasypus novemcinctus*
Baboon	*Papio hamadryas*
Bonobo	*Pan paniscus*
Bushbaby	*Otolemur garnettii*
Cat	*Felis catus*
Chimpanzee	*Pan troglodytes*
Cow	*Bos taurus*
Crab-eating macaque	*Macaca fascicularis*
Degu	*Octodon degus*
Dog	*Canis lupus familiaris*
Dolphin	*Tursiops truncatus*
Elephant	*Loxodonta africana*
Ferret	*Mustela putorius furo*
Gibbon	*Nomascus leucogenys*
Goat	*Capra hircus*
Golden hamster	*Mesocricetus auratus*
Gorilla	*Gorilla gorilla*
Ground squirrel	*Spermophilus citellus*
Guinea Pig	*Cavia porcellus*
Hedgehog	*Erinaceus europaeus*
Horse	*Equus caballus*
Human	*Homo sapiens*
Hyrax	*Procavia capensis*
Kangaroo rat	*Dipodomys ordii*
Killer whale	*Orcinus orca*
Lesser hedgehog tenrec	*Echinops telfairi*
Lesser egyptian jerboa	*Jaculus jaculus*
Manatee	*Trichechus inunguis*
Marmoset	*Callithrix jacchus*
Megabat	*Pteropus vampyrus*
Microbat	*Myotis lucifugus*
Mouse	*Mus musculus*
Mouse Lemur	*Microcebus murinus*
Naked mole rat	*Heterocephalus glaber*
Olive Baboon	*Papio anubis*
Opossum	*Monodelphis domestica*
Orangutan	*Pongo abelii*
Panda	*Ailuropoda melanoleuca*
Pig	*Sus scrofa*
Pika	*Ochotona princeps*
Platypus	*Ornithorhynchus anatinus*
Prairie vole	*Microtus ochrogaster*
Rabbit	*Oryctolagus cuniculus*
Rat	*Rattus norvegicus*
Rhesus macaque	*Macaca mulatta*
Rhinoceros	*Ceratotherium simum*
Sheep	*Ovis aries*
Shrew	*Sorex araneus*
Sloth	*Choloepus hoffmanni*
Squirrel	*Ictidomys tridecemlineatus*
Squirrel monkey	*Saimiri boliviensis*
Star-nosed mole	*Condylura cristata*
Tarsier	*Tarsius syrichta*
Tasmanian devil	*Sarcophilus harrisii*
Tree Shrew	*Tupaia belangeri*
Wallaby	*Macropus eugenii*
Walrus	*Odobenus rosmarus*

Supplementary Table S3. Likelihood ratio test statistics for models of variable selective pressure among sites (F3x4 and F61 codon frequency model).

Gene/LRT model	F3x4			F61		
	-2ΔlnL	*p* value (corrected *p* value)	% of sites (average dN/dS)	-2ΔlnL	*p* value (corrected *p* value)	% of sites (average dN/dS)
MGAM						
**Region1** (1-682aa)						
M1a vs M2a	43.27	$4.02x10^{-10}$ ($6.43x10^{-9}$)	2.8% (2.8)	39.45	$2.71x10^{-9}$ ($4.34x10^{-8}$)	2.8% (2.75)
M7 vs M8	61.99	$3.46x10^{-14}$ ($5.54x10^{-13}$)	4.4% (2.1)	54.67	$1.56x10^{-12}$ ($2.50x10^{-11}$)	4.1% (2.06)
**Region2** (683-1854aa)						
M1a vs M2a	49.72	$1.59x10^{-11}$ ($2.54x10^{-10}$)	1.8% (2.4)	54.07	$1.81x10^{-12}$ ($2.90x10^{-11}$)	1.6% (2.48)
M7 vs M8	74.72	$5.94x10^{-17}$ ($9.50x10^{-16}$)	6.6% (1.4)	73.31	$1.20x10^{-16}$ ($1.92x10^{-15}$)	4.7% (1.60)
SI						
**Region1** (1-1622aa)						
M1a vs M2a	196.24	$2.43x10^{-43}$ ($3.88x10^{-43}$)	2.2% (2.8)	215.67	$1.47x10^{-47}$ ($2.35x10^{-46}$)	2.9% (2.60)
M7 vs M8	219.59	$2.07x10^{-48}$ ($3.31x10^{-47}$)	3.3% (2.1)	248.24	$1.25x10^{-54}$ ($2.00x10^{-53}$)	5.8% (1.92)
**Region2** (1623-1833aa)						
M1a vs M2a	87.18	$1.43x10^{-19}$ ($2.29x10^{-18}$)	9.0% (2.8)	102.04	$6.96x10^{-23}$ ($1.11x10^{-21}$)	8.6% (2.81)
M7 vs M8	75.58	$3.87x10^{-17}$ ($6.19x10^{-16}$)	10.0% (2.1)	97.79	$5.83x10^{-22}$ ($9.33x10^{-21}$)	10.4% (2.22)
TREH						
**Region1** (1-315aa)						
M1a vs M2a	38.28	$4.86x10^{-9}$ ($7.78x10^{-8}$)	2.4% (3.1)	23.19	$9.20x10^{-6}$ ($1.47x10^{-4}$)	2.5% (2.43)
M7 vs M8	52.11	$4.83x10^{-12}$ ($7.72x10^{-11}$)	3.0% (2.1)	69.31	$8.89x10^{-16}$ ($1.42x10^{-14}$)	4.6% (1.8)
**Region2** (316-583aa)						
M1a vs M2a	0	1 (1)	11.0% (1.0)	-	-	-
M7 vs M8	14.1	0.0009 (0.014)	1.8% (2.1)	-	-	-
LCT						
M1a vs M2a	15.06	0.0005 (0.008)	1.2% (2.0)	17.85	0.0001 (0.0016)	1.1% (2.01)
M7 vs M8	96.07	$1.37x10^{-21}$ ($2.19x10^{-20}$)	7.4% (1.3)	82.71	$1.1x10^{-18}$ ($1.76x10^{-17}$)	6.4% (1.31)
SLC2A2						
M1a vs M2a	21.3	$2.36x10^{-5}$ ($3.78x10^{-4}$)	21.8% (2.5)	11.92	0.003 (0.048)	1.7% (2.18)
M7 vs M8	37.91	$5.86x10^{-9}$ ($9.38x10^{-8}$)	3.5% (1.8)	31.49	$1.45x10^{-7}$ ($2.32x10^{-6}$)	4.5% (1.52)

Note: M1a is a nearly neutral model that assumes one dN/dS class between 0 and 1 and one class with dN/dS=1; M2a (positive selection model) is the same as M1a plus an extra class of dN/dS >1. M7 (null model) assumes that 0<dN/dS<1 is β-distributed among sites in 10 classes; M8 (selection model) has an extra class with dN/dS >=1. 2ΔlnL is twice the difference of the natural logs of the maximum likelihood of the models being compared; p value is the p value of rejecting the neutral models (M1a or M8) in favor of the positive selection model (M2a or M8). % of sites (average dN/dS) is the estimated percentage of sites evolving under positive selection by M8 and M2a (dN/dS for these codons). 16 test were performed (9 genes, with 5 genes analyzed in two halves, and 1 gene in 3 subregions), and a Bonferroni correction was applied to *p* values.

Supplementary Table S4. Likelihood ratio test statistics for models of variable selective pressure among branches (M0 versus M1 models).

Gene	Degrees of freedom	-2ΔlnL	*p* value
MGAM			
**Region1** (1-682aa)	83	148.20	$1.44x10^{-5}$
**Region2** (683-1854aa)	83	234.72	$2.05x10^{-16}$
SI			
**Region1** (1-1622aa)	79	376.01	$3.1x10^{-40}$
**Region2** (1623-1833aa)	79	134.34	0.0001
TREH			
**Region1** (1-315aa)	83	111.37	0.02
LCT			
	86	182.38	$8.83x10^{-10}$
SLC2A2			
	89	236.2	$2.80x10^{-15}$

Note: M0 and M1 are free-ratio models that assume all branches to have the same dN/dS (M0) or allow each branch to have its own dN/dS (M1).

Supplementary Table S5. Single branch analysis. Results for the branch-site likelihood ratio tests are reported (significant branches only).

Gene	Foreground branch (MA versus MA1)	-2ΔlnL	*p* value (corrected *p* value)[a]	MEME-BEB sites[b]
MGAM				
**Region1**	Microbat	15.68	$7.51x10^{-5}$ ($9.38x10^{-5}$)	235R
**Region2**	Squirrel Monkey	18.51	$1.69x10^{-6}$ ($2.81x10^{-5}$)	1494E
	Macaque	42.85	$5.92x10^{-11}$ ($2.96x10^{-10}$)	1742T
	Olive Baboon	7.65	0.0057 ($5.66x10^{-3}$)	-
	Golden Hamster	31.44	$2.06x10^{-8}$ ($5.15x10^{-8}$)	1034P, 1688I
SI				
**Region1**	Elephant	40.63	$1.84x10^{-10}$ ($6.9x10^{-10}$)	606T
	Squirrel Monkey	20.84	$4.98x10^{-6}$ ($9.33x10^{-6}$)	810 R
	Macaque	14.17	0.0002 ($2.51x10^{-4}$)	-
	Tree Shrew	52.54	$4.21x10^{-13}$ (3.1610^{-12})	38V, 1105R, 1119H
	Node *Mouse-Rat*	30.61	$3.15x10^{-8}$ ($6.76x10^{-8}$)	35T, 180F, 482C, 1477K
	Mouse	30.61	$3.16x10^{-8}$ ($6.76x10^{-8}$)	483I, 676G, 990Y, 1602A
	Node *Pika-Rabbit*	13.41	0.0002 ($3.4x10^{-4}$)	92G
	Pig	41.52	$1.16x10^{-10}$ ($5.82x10^{-10}$)	297M, 298N, 299S
	Horse	18.32	$1.86x10^{-5}$ ($3.10x10^{-5}$)	180F, 1201T
	Microbat	32.75	$1.05x10^{-8}$ ($3.15x10^{-8}$)	359R, 1408N
	Megabat	52.54	$4.21x10^{-13}$ ($3.16x10^{-12}$)	560S, 1006A, 1201T, 1405T, 1449S
**Region2**	Squirrel	37.35	$9.85x10^{-10}$ ($8.86x10^{-9}$)	1735Y
	Horse	7.66	0.006 ($1.69x10^{-2}$)	1747L
	Microbat	11.17	0.0008 ($3.74x10^{-3}$)	1819E
TREH				
**Region1**	Microbat	33.67	$6.51x10^{-9}$ ($1.30x10^{-8}$)	26P, 28C, 287P
	Platypus	22.72	$1.88x10^{-6}$ ($1.88x10^{-6}$)	-
LCT				
	Elephant	15.1	0.0001 ($3.06x10^{-4}$)	244D, 947A, 1711I
	Chimpanzee	18.27	$1.92x10^{-5}$ ($8.63x10^{-5}$)	1411H, 1442L
	Tree Shrew	5.26	0.02($3.28x10^{-2}$)	-
	Dolphin	6.28	0.01 ($2.2x10^{-2}$)	-
	Cat	10.62	0.001 ($2.51x10^{-3}$)	-
SLC2A2				
	Ferret	24.71	$6.67x10^{-7}$	-

NOTE: MA and MA1 are branch-site models that assume four classes of sites: the MA model allows a proportion of codons to have $dN/dS \geq 1$ on the foreground branches, whereas the MA1 model does not. 2ΔlnL is twice the difference of the natural logs of the maximum likelihood of the models being compared.

[a] *p* values were FDR-corrected for the number of tested branches (see also Figures S1 and S2)

[b] positively selected sites identified by both BEB and MEME

Supplementary Table S6. Positively selected sites in the human,chimpanzee and gorilla lineages.

Gene	Lineage	Codon	Ancestral AA	Human/chimpanzee AA	Pr[a]	dbSNP	Other methods[b]
LCT							
	Human	408	Ala	Val	0.942	rs185361924	
		426	Pro	Leu	0.969	-	
		444	Arg	Cys	0.952	rs148438204	
	Chimpanzee	1677	Phe	Tyr	0.865	-	
		1694	Ala	Asp	0.859	-	
SI							
	Human	1772	Ile	Leu	0.906	-	
		1802	Ser	Thr	0.949	rs9917722	BEB and MEME
		1808	His	Arg	0.946	rs149654947	
	Chimpanzee	162	Ile	Val	0.804	-	
		178	Lys	Gln	0.822	-	MEME
		1709	Lys	Asn	0.857	-	
		1719	Met	Thr	0.853	-	
SLC5A1							
	Human	9	Thr	Lys	0.860	-	MEME
		312	Ala	Gly	0.900	rs142230209	
		341	Asp	Glu	0.903	-	
		411	Thr	Ala	0.852	rs17683430	
		493	Phe	Leu	0.829	-	
		615	Gln	His	0.906	rs33954001	MEME
		645	Met	Leu	0.913	-	MEME
TAS1R2							
	Chimpanzee	242	Thr	Ala	0.809	-	
		280	Thr	Ser	0.811	-	
TAS1R3							
	Chimpanzee	441	Pro	Met	0.853	-	
TREH							
	Gorilla	297	Ala	Ser	0.809	-	MEME

[a] Posterior probability of $\gamma > 0$ as detected by gammaMap.
[b] Other methods that identified the same codon as positively selected

Supplementary Table S7. Nucleotide diversity and Tajima's D for the 9 brush-border genes.

Gene	Population	S[a]	Θ_W (x 10^{-4})		Π (x 10^{-4})		Tajima's D	
			value	rank[b]	value	rank[b]	value	rank[b]
MGAM	YRI	652	11.005	0.95	11.888	0.91	0.269	0.54
	CEU	383	6.444	0.84	8.109	0.80	0.86	0.54
	CHBJPT	345	5.805	0.88	8.021	0.84	1.27	0.48
SI	YRI	346	6.501	0.54	9.111	0.77	1.34	0.94
	CEU	221	4.139	0.43	6.26	0.61	1.693	0.85
	CHBJPT	177	3.315	0.48	3.753	0.33	0.434	0.22
TREH	YRI	67	5.848	0.43	5.666	0.37	-0.099	0.32
	CEU	41	3.567	0.30	5.621	0.53	1.768	0.86
	CHBJPT	39	3.393	0.49	6.105	0.67	2.444	0.87
SLC2A5	YRI	135	7.683	0.71	8.715	0.73	0.44	0.64
	CEU	96	5.446	0.69	7.069	0.71	0.963	0.58
	CHBJPT	95	5.389	0.85	7.139	0.77	1.048	0.40
SLC5A1	YRI	132	3.529	0.90	2.63	0.05	-0.835	0.06
	CEU	176	4.691	0.55	3.132	0.20	-1.093	0.05
	CHBJPT	47	1.253	0.05	0.934	0.03	-0.789	0.05
SLC2A2	YRI	122	7.453	0.67	10.074	0.83	1.149	0.91
	CEU	77	4.689	0.55	4.902	0.42	0.145	0.28
	CHBJPT	52	3.167	0.43	1.988	0.12	-1.164	0.03
TAS1R2	YRI	152	14.178	0.99	18.446	0.99	0.99	0.89
	CEU	112	10.414	0.99	16.066	0.98	1.763	0.86
	CHBJPT	90	8.368	0.98	13.241	0.97	1.876	0.70
TAS1R3	YRI	1	0.454	0.01	0.082	<0.01	-0.911	0.05
	CEU	2	0.906	0.01	0.277	0.01	-1.032	0.06
	CHBJPT	2	0.906	0.03	0.878	0.03	-0.046	0.13

[a] number of segregating sites;
[b] percentile rank relative to a distribution of ~1000 randomly selected genes.

References

1000 Genomes Project Consortium, et al. 2010. A map of human genome variation from population-scale sequencing. Nature 467:1061-1073.

Anisimova M., J. P. Bielawski, and Z. Yang. 2002. Accuracy and power of bayes prediction of amino acid sites under positive selection. Mol. Biol. Evol. 19:950-958.

Barreiro L. B., et al. 2009. Evolutionary dynamics of human Toll-like receptors and their different contributions to host defense. PLoS Genet. 5:e1000562.

Capella-Gutierrez S., J. M. Silla-Martinez, and T. Gabaldon. 2009. trimAl: a tool for automated alignment trimming in large-scale phylogenetic analyses. Bioinformatics 25:1972-1973.

Cereda M., M. Sironi, M. Cavalleri, and U. Pozzoli. 2011. GeCo++: a C++ library for genomic features computation and annotation in the presence of variants. Bioinformatics 27:1313-1315.

Delport W., A. F. Poon, S. D. Frost, and S. L. Kosakovsky Pond. 2010. Datamonkey 2010: a suite of phylogenetic analysis tools for evolutionary biology. Bioinformatics 26:2455-2457.

Fay J. C., and C. I. Wu. 2000. Hitchhiking under positive Darwinian selection. Genetics 155:1405-1413.

Guindon S., F. Delsuc, J. F. Dufayard, and O. Gascuel. 2009. Estimating maximum likelihood phylogenies with PhyML. Methods Mol. Biol. 537:113-137.

Karolchik D., et al. 2004. The UCSC Table Browser data retrieval tool. Nucleic Acids Res. 32:D493-6.

Kosakovsky Pond S. L., and S. D. Frost. 2005. Not so different after all: a comparison of methods for detecting amino acid sites under selection. Mol. Biol. Evol. 22:1208-1222.

Kosakovsky Pond S. L., D. Posada, M. B. Gravenor, C. H. Woelk, and S. D. Frost. 2006. Automated phylogenetic detection of recombination using a genetic algorithm. Mol. Biol. Evol. 23:1891-1901.

Kosakovsky Pond S. L., et al. 2011. A random effects branch-site model for detecting episodic diversifying selection. Mol. Biol. Evol. 28:3033-3043.

Kuhn R. M., D. Haussler, and W. J. Kent. 2013. The UCSC genome browser and associated tools. Brief Bioinform 14:144-161.

Murrell B., et al. 2012. Detecting individual sites subject to episodic diversifying selection. PLoS Genet. 8:e1002764.

Nei M., and W. H. Li. 1979. Mathematical model for studying genetic variation in terms of restriction endonucleases. Proc. Natl. Acad. Sci. U. S. A. 76:5269-5273.

Schaffner S. F., et al. 2005. Calibrating a coalescent simulation of human genome sequence variation. Genome Res. 15:1576-1583.

Tajima F. 1989. Statistical method for testing the neutral mutation hypothesis by DNA polymorphism. Genetics 123:585-595.

Thornton K. 2003. Libsequence: a C++ class library for evolutionary genetic analysis. Bioinformatics 19:2325-2327.

Vilella A. J., et al. 2009. EnsemblCompara GeneTrees: Complete, duplication-aware phylogenetic trees in vertebrates. Genome Res. 19:327-335.

Watterson G. A. 1975. On the number of segregating sites in genetical models without recombination. Theor. Popul. Biol. 7:256-276.

Wernersson R., and A. G. Pedersen. 2003. RevTrans: Multiple alignment of coding DNA from aligned amino acid sequences. Nucleic Acids Res. 31:3537-3539.

Wilson D. J., R. D. Hernandez, P. Andolfatto, and M. Przeworski. 2011. A population genetics-phylogenetics approach to inferring natural selection in coding sequences. PLoS Genet. 7:e1002395.

Wright S. 1950. Genetical structure of populations. Nature 166:247-249.

Yang Z. 2007. PAML 4: phylogenetic analysis by maximum likelihood. Mol. Biol. Evol. 24:1586-1591.

Yang Z., W. S. Wong, and R. Nielsen. 2005. Bayes empirical bayes inference of amino acid sites

under positive selection. Mol. Biol. Evol. 22:1107-1118.
Zeng K., Y. X. Fu, S. Shi, and C. I. Wu. 2006. Statistical tests for detecting positive selection by utilizing high-frequency variants. Genetics 174:1431-1439.
